# Parametric modeling of random polygonal aggregates in concrete and nusssmerical simulation of uniaxial compression using SPH

**DOI:** 10.1038/s41598-026-58544-5

**Published:** 2026-06-23

**Authors:** Xiaonian Chen, Yiwei Gao, Xiuchang Song, Zhengxiong Bai, Zhen Li, Daniel Dias

**Affiliations:** 1 Yellow River Engineering Consulting Co., Ltd, Zhengzhou, 450003 China; 2 Key Laboratory of Water Management and Water Security for Yellow River Basin (Under Construction), Zhengzhou, China; 3 College of Civil and Transportation Engineering, Shenzhen University, Shenzhen, 518060 China

**Keywords:** SPH, Concrete, Random polygonal aggregate, Numerical simulation, Uniaxial compression, Engineering, Materials science, Mathematics and computing

## Abstract

Random polygonal aggregates represent the most realistic aggregate morphology in the mesostructure of concrete. Their angular characteristics and spatial distribution have a decisive influence on the stress concentration, crack initiation, and ultimate failure mode of the material under uniaxial compression. Therefore, in-depth research in this area is essential for revealing the essence of the macroscopic mechanical behavior of concrete. To this end, this paper presents an SPH-based simulation framework for uniaxial compression failure of concrete by integrating the Mohr–Coulomb criterion with tensile cutoff and an improved smoothing kernel function. A key component is the development of an in-house particle generation program for random polygonal aggregates, which achieves automatic generation satisfying geometric constraints and precise particle attribute classification based on the separating axis theorem and the ray casting method. Through three sets of comparative numerical examples, the effects of the number of aggregates (30, 40, 50), aggregate size range (1–8 mm, 1–10 mm, 1–12 mm), and shape complexity (number of edges: 8–15, 8–20, 8–25) on the failure mode of concrete are systematically investigated. The findings reveal that an increase in aggregate density or expansion of the size range promotes a transition from localized shear failure to more distributed damage patterns, accompanied by enhanced material brittleness. In contrast, increasing the shape complexity of aggregates (i.e., a larger number of edges) shifts the failure mode from “angularity-dominated concentrated shear failure” to “interface-dominated fine diffuse failure,” significantly improving material ductility. Comparison and validation with existing results show that the proposed method achieves good agreement in terms of failure morphology and crack evolution. This study provides an efficient modeling approach and theoretical support for the mesomechanical analysis of concrete with irregular aggregates.

## Introduction

Concrete is the most widely used artificial construction material in contemporary civil engineering. Its macroscopic mechanical properties, particularly the strength and deformation characteristics under uniaxial compression, serve as the core basis for structural design, safety assessment, and durability prediction^1–3^. The essence of the macroscopic mechanical behavior of concrete originates from its complex mesostructural characteristics. From a mesoscopic perspective, concrete is not a homogeneous material but a multiphase heterogeneous composite composed of aggregates, cement matrix, and other constituents^4–6^. Among these phases, aggregates act as the primary load-bearing component, and their geometric characteristics (e.g., shape, size, gradation, and spatial distribution) couple with the mechanical properties of the matrix, collectively determining the stress distribution, damage evolution path, and ultimate macroscopic failure mode of concrete under external loading^7,8^. Compared with circular aggregates, the aggregate morphology in real concrete often exhibits irregular polygonal characteristics, and their angular features and convex-concave contours have a decisive influence on local stress concentration and crack initiation^9–15^. Therefore, an in-depth investigation into the mesoscopic failure mechanism of concrete with random polygonal aggregates under uniaxial compression not only helps to reveal its own mechanical behavior but also provides important references for mesomechanical studies of other types of concrete.

To reveal the compressive failure mechanism of concrete, researchers have mainly adopted two approaches: experimental studies and numerical simulations. At the experimental level, traditional physical tests can provide macroscopic mechanical response data but struggle to capture the crack initiation and propagation processes inside the material in real time and intuitively^7,13,15–17^[. In recent years, the application of advanced testing techniques such as digital image correlation (DIC) and acoustic emission (AE) has made it possible to analyze crack evolution from the perspectives of surface strain fields and internal damage signals^18,19^; however, these methods still cannot clearly and completely present the entire damage evolution process of concrete mesostructures^19^.

In the field of numerical simulation, accurate characterization of aggregate shape is a key step in constructing realistic concrete models. Early studies mostly adopted simplified models using circular or elliptical aggregates^9,11,12,14,20^. Although such models are convenient for generation and computation, they fail to reflect the angular effects and interlocking actions of real aggregates, leading to deviations in the prediction of crack propagation paths. To overcome this limitation, researchers have recently developed modeling techniques for polygonal and arbitrarily shaped aggregates^21,22^. However, the introduction of polygonal aggregates makes the geometric compatibility between aggregates and the matrix more complex while imposing higher demands on the ability of numerical methods to simulate crack propagation.

Addressing these challenges, researchers have developed various numerical methods applicable to mesoscale concrete analysis^23,24^. The finite element method (FEM) is currently the most widely used computational framework, and several mature fracture simulation techniques have been derived from it. The cohesive zone model introduces cohesive elements and corresponding traction–separation constitutive relations to accurately simulate crack initiation and progressive propagation without artificially constructing crack tip singular fields^25,26^. Owing to its clear physical meaning and ease of implementation, it has become a mainstream method for fracture analysis of quasi-brittle materials. The extended finite element method (XFEM) employs enrichment functions to describe displacement discontinuities across cracks, enabling cracks to propagate through elements without mesh regeneration, and exhibits strong capabilities in simulating branching and tortuous cracks^27,28^. The phase-field method, as an independent fracture analysis theory, smears sharp crack geometries into continuously distributed phase-field variables and automatically tracks crack initiation, propagation, and branching by solving governing equations^29^. This method does not rely on pre-defined crack paths and maintains good numerical stability under complex crack evolution scenarios, and has been widely applied to concrete fracture simulation. Different from fracture-based methods, nonlocal damage models approach the problem from the perspective of damage mechanics, coupling strain information from material point neighborhoods to perform damage evolution calculations^30^, significantly mitigating the mesh sensitivity issues of local damage models in the strain-softening stage and achieving mesh-independent numerical results^31,32^. Overall, all the above methods can simulate the crack evolution process in concrete and have laid a solid foundation for understanding its fracture mechanisms.

However, when these methods are applied to mesoscale concrete models containing random polygonal aggregates, several difficulties arise. First, the complex interfaces between polygonal aggregates and the matrix require extremely fine mesh generation to ensure geometric compatibility, which not only significantly increases computational cost but also readily leads to mesh distortion problems. Second, the above methods are all constrained to varying degrees by mesh topology when simulating crack propagation: the cohesive zone model requires pre-definition of crack paths; XFEM encounters numerical difficulties when handling multiple intersecting cracks; the phase-field method is sensitive to the selection of phase-field regularization parameters; and nonlocal damage models still exhibit some mesh dependence in the post-peak softening stage. In addition to FEM, researchers have also attempted other numerical methods. The discrete element method (DEM) simulates inter-particle contact and fracture using particle units and can handle aggregate shape complexity^33,34^; however, its calibration process for computational parameters is cumbersome, and it struggles to accurately simulate fracture within aggregates. Peridynamics (PD), as a nonlocal theory, can naturally simulate the failure process from continuous to discontinuous states and has shown potential in concrete fracture simulation^35^; nevertheless, its model construction is mostly based on homogenization assumptions or simplified geometries, lacking the ability to accurately characterize the complex geometric morphology of random polygonal aggregates, and its computational cost is high. Therefore, existing numerical methods for simulating concrete with random polygonal aggregates are either limited by the accuracy of aggregate shape characterization or struggle to accurately simulate the initiation and evolution of complex cracks. There is an urgent need for a novel computational approach that combines the capability of complex geometric modeling with the advantages of meshless fracture simulation.

Smoothed particle hydrodynamics (SPH) is a purely meshless Lagrangian particle method. It possesses both the natural advantages of meshless methods in handling large deformation and fracture problems and the flexibility of particle methods in simulating crack initiation, propagation, and material failure without the need for mesh reconstruction^36–39^. Compared with the finite element method, SPH avoids mesh distortion and remeshing issues; compared with the discrete element method, its governing equations are more intuitive; and compared with peridynamics, SPH has higher computational efficiency. However, when applying SPH to mesoscale failure simulation of concrete, the core challenge lies in how to accurately and efficiently construct an initial computational model that reflects the distribution characteristics of random polygonal aggregates^18,40^. Although some studies have applied SPH to fracture simulation of concrete materials, their computational models are mostly limited to simplified scenarios of homogeneous materials or those containing only a few preset cracks, failing to reflect the realistic mesostructural characteristics of concrete^41^. More critically, there is currently a lack of a method that can automatically generate a particle model of random polygonal aggregates satisfying complex geometric constraints based on user-defined geometric parameters (e.g., number of aggregates, size range, edge number range, minimum spacing). This severely restricts the widespread application and further development of the SPH method in mesomechanics research of concrete.

In view of this, this paper proposes a simulation method for uniaxial compression failure of concrete with random polygonal aggregate models based on the SPH method. The main contributions of this work are a framework demonstrating the following capabilities: (i) seamless integration of SPH with a parametric random polygonal aggregate generator that satisfies complex geometric constraints; (ii) adoption of a binary fracture model that naturally captures sharp crack surfaces without mesh reconstruction; and (iii) efficient parametric analysis of aggregate geometric effects (density, size distribution, and shape complexity). This combination of capabilities has not been systematically applied to the study of aggregate geometry effects in mesoscale damage analysis of concrete.

In terms of specific implementation, first, the Mohr–Coulomb criterion with tensile cutoff is introduced as the particle failure criterion, and an improved smoothing kernel function is constructed to enable spontaneous crack initiation and propagation within the SPH framework. Second, to address the modeling difficulty of complex random aggregate models, this paper independently develops a parametric particle generation method for random polygonal aggregates. This method achieves accurate collision detection of polygonal aggregates based on the separating axis theorem and can automatically generate an SPH particle computational model with separate aggregate and matrix phases based on user-defined computational domain, number of aggregates, size range, edge number range, and spacing constraints, realizing an integrated construction process from aggregate geometry definition to SPH computational model. Based on this method, multiple sets of comparative numerical examples are designed to systematically investigate the effects of aggregate number, size distribution range, and aggregate shape complexity (range of edge numbers) on the failure mode, crack evolution path, and macroscopic mechanical response of concrete specimens under uniaxial compression. The aim is to reveal the regulatory role of mesoscopic geometric characteristics of random polygonal aggregates on the failure mechanism of concrete and to provide a reliable and efficient new approach for the application of the SPH method in the field of concrete mesomechanics.

## Fundamental principles of SPH and fracture treatment method

### Basic governing equations of SPH

#### Governing equations

Each SPH particle is required to satisfy four fundamental equations, namely the continuity equation, the momentum equation, the energy equation, and the equation of motion^36^.

1 $$\left\{ {\begin{array}{*{20}c} {\frac{{d\rho_{i} }}{dt} = \sum\limits_{j = 1}^{N} {m_{j} v_{ij}^{\beta } \frac{{\partial W_{ij,\beta } }}{{\partial x_{i}^{\beta } }}} } \\ {\frac{{dv_{i}^{\alpha } }}{dt} = \sum\limits_{j = 1}^{N} {m_{j} (\frac{{\sigma_{i}^{\alpha \beta } }}{{\rho_{i}^{2} }} + \frac{{\sigma_{j}^{\alpha \beta } }}{{\rho_{j}^{2} }} + T_{ij} )\frac{{\partial W_{ij,\beta } }}{{\partial x_{i}^{\beta } }}} } \\ {\frac{{de_{i} }}{dt} = \frac{1}{2}\sum\limits_{j = 1}^{N} {m_{j} (\frac{{\sigma_{i}^{\alpha \beta } }}{{\rho_{i}^{2} }} + \frac{{\sigma_{j}^{\alpha \beta } }}{{\rho_{j}^{2} }} + T_{ij} )v_{ij}^{\beta } \frac{{\partial W_{ij,\beta } }}{{\partial x_{i}^{\beta } }}} } \\ {\frac{{dx_{i}^{\alpha } }}{dt} = v_{i}^{\alpha } } \\ \end{array} } \right.$$

where *ρ* denotes the particle density (kg/m^3^), *t* is the computational time (s), *m* represents the particle mass (kg), *v* is the particle velocity (m/s), *x* is the particle position coordinate (m), *σ*^*αβ*^ is the normal stress tensor of the particle (Pa), *e* is the particle energy (J), *τ*^*αβ*^ denotes the shear stress components (Pa), *ε*^*αβ*^ represents the strain components, *T* is the artificial viscosity term introduced to suppress non-physical oscillations during the computation, and *W* is the smoothing kernel function used in the SPH method.

#### Elastic Equations

In the SPH method, the stress components of each particle are calculated using elastic constitutive equations. The total stress component *σ*^*αβ*^ consists of the hydrostatic pressure *p* and the shear stress *τ*, and can be expressed as^36,42^:

2 $$\sigma^{\alpha \beta } = { - }p\delta^{\alpha \beta } + \tau^{\alpha \beta }$$

The hydrostatic pressure *p* can be estimated using an equation of state, which is expressed as:

3 $$p = (1 - \frac{1}{2}\Gamma \eta )p_{H} + \Gamma \rho e$$

where *p*_*H*_ denotes the Hugoniot curve function, and *Γ* is the Gruneisen parameter. The elastic constitutive equations for solids calculate the variation of the stress rate based on strain and strain rate, and are expressed as follows:

4 $$\overset{\lower0.5em\hbox{$\smash{\scriptscriptstyle\frown}$}}{\tau}_{\alpha \beta } = B(\varepsilon^{\alpha \beta } - \frac{1}{3}\delta^{\alpha \beta } \varepsilon^{\gamma \gamma } ) + \tau^{\alpha \gamma } R^{\beta \gamma } + \tau^{\gamma \beta } R^{\alpha \gamma }$$

where $$\overset{\lower0.5em\hbox{$\smash{\scriptscriptstyle\frown}$}}{\tau }$$ denotes the stress rate tensor with the unit of Pa/s, *B* represents the shear modulus with the unit of Pa, and *R* denotes the spin rate tensor, which can be defined as follows:

5 $$R^{\alpha \beta } = \frac{1}{2}\left( {\frac{{\partial v^{\alpha } }}{{\partial x^{\beta } }} - \frac{{\partial v^{\beta } }}{{\partial x^{\alpha } }}} \right)$$

### SPH fracture treatment

#### Particle failure treatment method

**Fig. 1:**
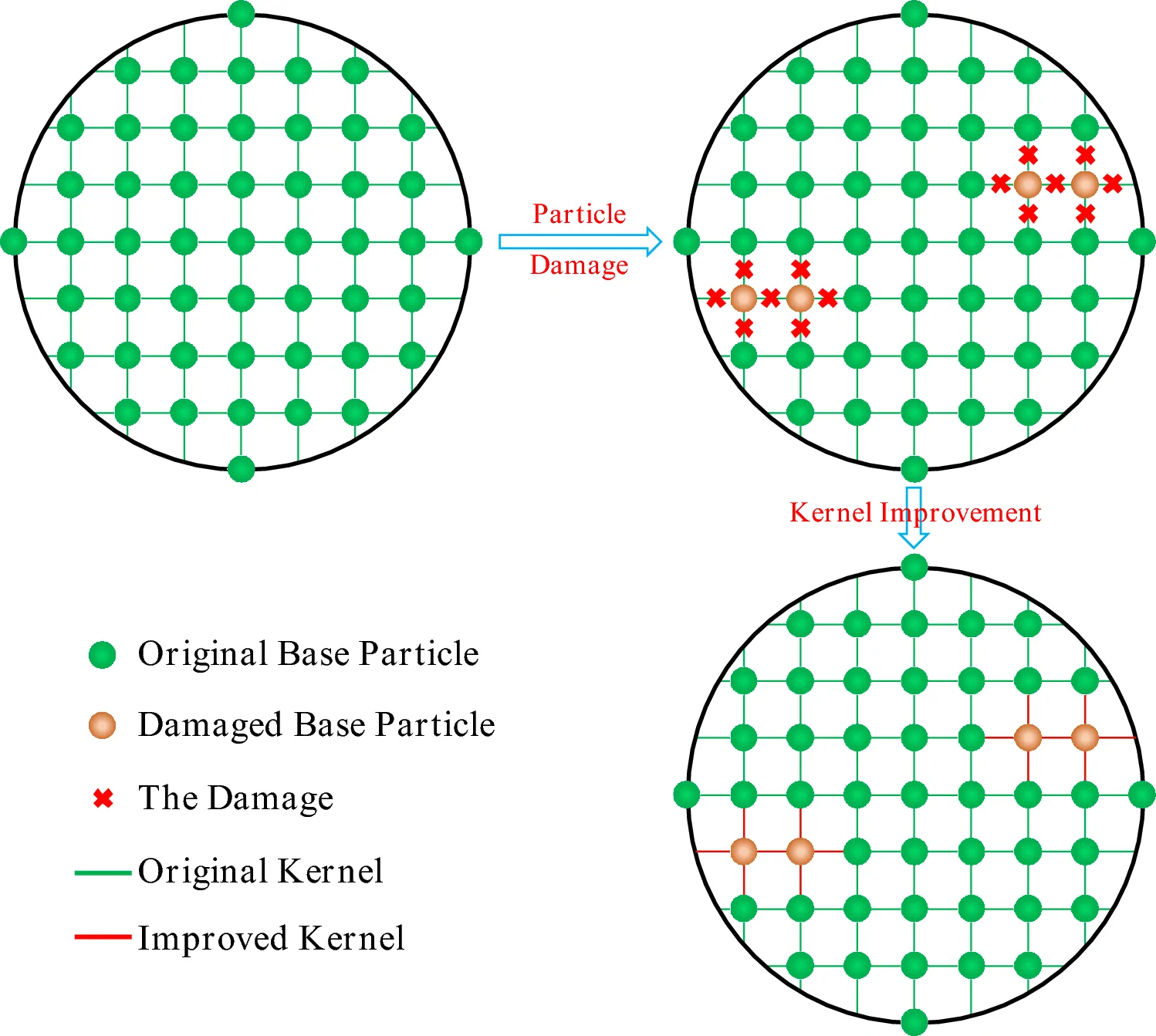
Schematic illustration of SPH particle failure.

As shown in Eq. (1), for each particle, the SPH method transfers physical quantities between particles through the derivative ${{\partial W_{ij,\beta } } \mathord{\left/ {\vphantom {{\partial W_{ij,\beta } } {\partial x_{i}^{\beta } }}} \right. \kern-0pt} {\partial x_{i}^{\beta } }}$ of the smoothing kernel function, including density, velocity, energy, and position. By appropriately modifying the derivative of the smoothing kernel function and cutting off the interactions between particles, particle failure behavior can be represented. Based on this concept, a fracture indicator *ξ* is introduced to characterize particle damage. The particle failure process is illustrated in Fig. 1. First, whether a particle is damaged is determined. If particle failure occurs, *ξ* = 0; otherwise, *ξ* = 1. Accordingly, a modified kernel function *D* that considers fracture damage is defined, which can be expressed as:

6 $$\frac{{\partial D_{ij,\beta } }}{{\partial x_{i}^{\beta } }} = \xi_{i} \cdot \frac{{\partial W_{ij,\beta } }}{{\partial x_{i}^{\beta } }}$$

Therefore, the SPH governing equations in the improved form can be expressed as:

7 $$\left\{ {\begin{array}{*{20}c} {\frac{{d\rho_{i} }}{dt} = \sum\limits_{j = 1}^{N} {m_{j} v_{ij}^{\beta } \frac{{\partial D_{ij,\beta } }}{{\partial x_{i}^{\beta } }}} } \\ {\frac{{dv_{i}^{\alpha } }}{dt} = \sum\limits_{{j \in {\mathrm{S}}}}^{N} {m_{j} (\frac{{\sigma_{i}^{\alpha \beta } }}{{\rho_{i}^{2} }} + \frac{{\sigma_{j}^{\alpha \beta } }}{{\rho_{j}^{2} }} + T_{ij} )\frac{{\partial D_{ij,\beta } }}{{\partial x_{i}^{\beta } }} + \sum\limits_{{j \in {\mathrm{W}}}}^{N} {m_{j} (\frac{{\sigma_{i}^{\alpha \beta } }}{{\rho_{i}^{2} }} + \frac{{\sigma p_{{}}^{\alpha \beta } }}{{\rho_{j}^{2} }} + T_{ij} )\frac{{\partial D_{ij,\beta } }}{{\partial x_{i}^{\beta } }} + } } } \\ {\frac{{de_{i} }}{dt} = \frac{1}{2}\left( {\sum\limits_{{j \in {\mathrm{S}}}}^{N} {m_{j} (\frac{{\sigma_{i}^{\alpha \beta } }}{{\rho_{i}^{2} }} + \frac{{\sigma_{j}^{\alpha \beta } }}{{\rho_{j}^{2} }} + T_{ij} )v_{ij}^{\beta } \frac{{\partial D_{ij,\beta } }}{{\partial x_{i}^{\beta } }} + \sum\limits_{{j \in {\mathrm{W}}}}^{N} {m_{j} (\frac{{\sigma_{i}^{\alpha \beta } }}{{\rho_{i}^{2} }} + \frac{{\sigma p_{{}}^{\alpha \beta } }}{{\rho_{j}^{2} }} + T_{ij} )v_{ij}^{\beta } \frac{{\partial D_{ij,\beta } }}{{\partial x_{i}^{\beta } }}} } } \right)} \\ {\frac{{dx_{i}^{\alpha } }}{dt} = v_{i}^{\alpha } } \\ \end{array} } \right.$$

In this study, a binary fracture model is employed to describe particle failure behavior. When a particle satisfies the Mohr–Coulomb criterion or the tensile cutoff criterion, the fracture factor *ξ* is directly set to zero, completely terminating the interaction between that particle and its neighbors. The fractured particle no longer participates in subsequent momentum and energy exchanges, and no progressive damage evolution stage is incorporated in this process.

This approach effectively captures the brittle fracture characteristics of concrete under uniaxial compression, generating clearly defined crack surfaces and failure paths, which facilitates comparative analyses of the influence of aggregate geometric parameters on failure modes. However, this model cannot represent the progressive stiffness degradation and microcrack accumulation prior to macroscopic failure of the specimen, leading to certain deviations between the simulated curves and experimental results. In the pre-peak stage, due to the absence of gradual microcrack development and continuous stiffness deterioration, the stress–strain curve exhibits an approximately linear elastic rise, failing to reflect the nonlinear hardening characteristics of real concrete. In the post-peak stage, as a large number of particles fail simultaneously after the coalescence of major cracks, the load-bearing capacity drops abruptly, resulting in a steep descending curve that differs from the gradual softening behavior observed in experiments.

Overall, this simplified model offers clear advantages. On the one hand, it allows for the identification of crack propagation paths and failure morphologies without interference, while reducing computational complexity and ensuring computational efficiency across multiple cases. On the other hand, since all cases in this study are analyzed under the same modeling rules, the observed trends and core conclusions regarding the influence of aggregate geometric parameters on concrete failure behavior are fully reliable.

#### Fracture criterion

In this study, the Mohr–Coulomb criterion with a tensile cutoff is adopted as the fracture criterion. This criterion has been widely used in previous numerical simulations of fracture mechanics and has demonstrated good applicability. The corresponding expression can be written as:

8 $$\sigma_{f} = \sigma_{t}$$

9 $$\tau_{f} = c + \sigma_{f} {\mathrm{tan}}\phi$$

where *σ*_*f*_ and *τ*_*f*_ denote the maximum tensile stress and shear stress acting on the hypothetical failure plane, respectively; *σ*_*t*_ is the tensile strength of the material; *c* is the material cohesion; and *φ* is the internal friction angle of the material.

It should be noted that the implementation of the fracture criterion follows the same binary philosophy described in “Particle failure treatment method” section. Specifically, when the tensile condition (*σ*_*f*_ ≥ *σ*_*t*_) or the shear condition (*τ*_*f*_ ≥ *c*—*σ*_*f*_ tan*φ*) is met, the particle is immediately marked as failed, with *ξ* = 0, and no progressive degradation of material properties occurs. This modeling choice simplifies the fracture process and focuses on the initiation and propagation of macroscopic cracks, which is adequate for the parametric comparative study of aggregate geometric effects.

#### Computational procedure

Meshfree methods such as SPH exhibit clear advantages in simulating large deformation, moving boundaries, and complex fracture networks. However, the construction of the initial particle distribution remains challenging, particularly for problems involving complex geometries, multi-material interfaces, or accurately prescribed initial physical fields. In contrast, generating SPH initial particles based on a finite element (FEM) mesh can fully exploit the mature and accurate preprocessing capabilities of FEM in representing complex geometries and material interfaces, thereby efficiently producing an initial particle distribution that strictly conforms to actual boundaries and ensures geometric fidelity.

Moreover, this approach provides an effective means for high-accuracy initialization of physical field variables. By directly mapping the complete initial state variables, such as displacement, stress, and damage, from a solved FEM model to SPH particles, a mechanically balanced and physically meaningful initial condition can be established for subsequent simulations, such as fracture propagation analysis. This strategy effectively integrates the advantages of FEM and meshfree methods and significantly enhances the accuracy and reliability of the overall numerical simulation framework.

**Fig. 2:**
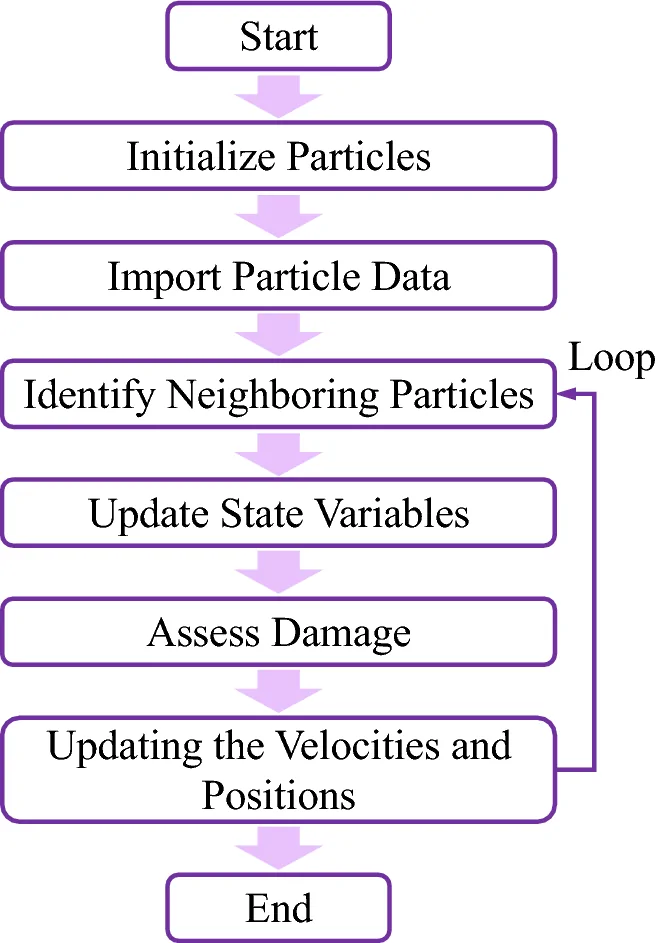
Flowchart of the SPH calculation.

Therefore, FEM is adopted in this study to generate the initial SPH particles. The computational flowchart is shown in Fig. 2.

1. Generate the initial particles, with no particles assigned in the fracture regions.
2. Import particle information, including particle coordinates, initial velocities, initial stresses, and boundary conditions.
3. Identify interacting particle pairs using a particle interaction search algorithm. In this study, a linked-list search method is employed, and the corresponding smoothing kernel functions are updated.
4. Update particle density, stress, and energy according to Eqs. (1) and (2).
5. Determine whether particle fracture damage occurs based on Eqs. (8) and (9).
6. Update particle velocities and positions. If the simulation has not been completed, return to step (3).

## Particle generation method for random polygonal aggregate models

To accurately simulate the mesoscopic mechanical behavior of random polygonal aggregates in concrete, it is necessary to construct a numerical model that reflects the randomness of its internal structure. To address the issues of single aggregate shape and extensive manual intervention in distribution in traditional modeling methods, this chapter proposes a particle generation method for random polygonal aggregate models, aiming to achieve fully automated modeling from parameter input to model output. In this method, the computational domain is first parameterized and a regular initial particle set is generated. Subsequently, a controlled random iterative algorithm and the separating axis theorem are adopted to generate random polygonal aggregates that satisfy geometric constraints and spacing requirements. Finally, based on the point-in-polygon inclusion criterion, the particles are automatically classified into "aggregate region particles" or "matrix region particles", forming a computational model that can be directly used for numerical simulation.

### Computational domain and initial particle generation

**Fig. 3:**
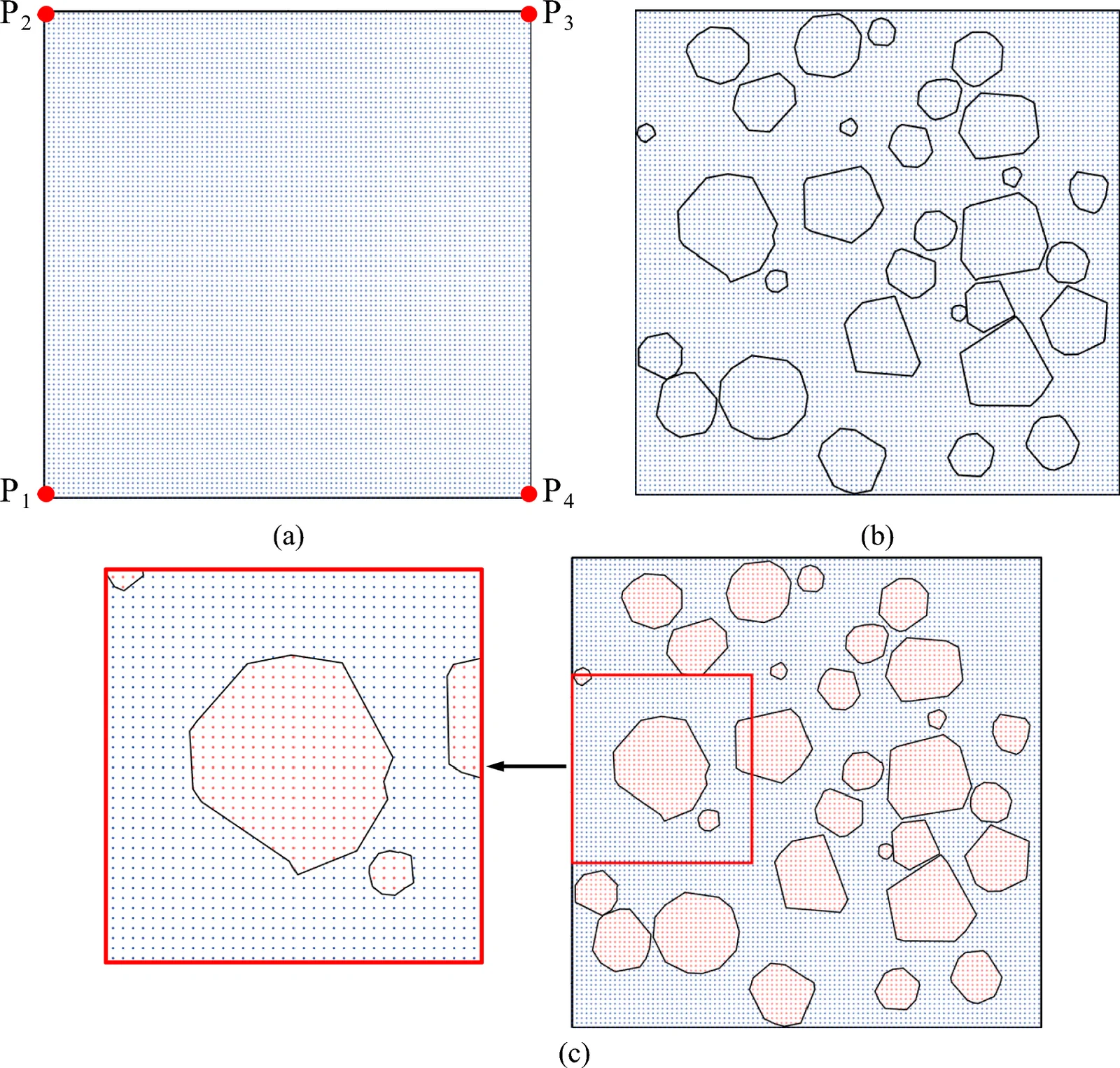
Schematic diagram of the construction of the random polygonal aggregate model: (**a**) computational domain and initial particles; (**b**) distribution of random polygonal aggregates; (**c**) classification results of aggregate and matrix particles.

The computational domain in this study is defined as a square region, which is parametrically described by the coordinates of its four vertices: bottom-left corner P_1_(*x*_min_, *y*_min_), top-left corner P_2_(*x*_min_, *y*_max_), top-right corner P_3_(*x*_max_, *y*_max_), and bottom-right corner P_4_(*x*_max_, *y*_min_), as shown in Fig. 3(a). Here, *x*_min_ and *x*_max_ are the minimum and maximum coordinate values of the computational domain in the X direction, respectively, and *y*_min_ and *y*_max_ are the minimum and maximum coordinate values in the Y direction, respectively.

To discretize the continuous computational domain into a set of particles suitable for numerical computation, a generation step size d_grid_ is introduced. Based on the boundary coordinates and the generation step size, the system generates equally spaced coordinate sequences in the X and Y directions, respectively:

X-axis coordinate sequence:

10 $$\left( {x_{1} ,x_{2} ,...,x_{m} } \right)\begin{array}{*{20}c} , & {} \\ \end{array} x_{i} = x_{\min } + \left( {i - 1} \right) \cdot d_{{{\mathrm{grid}}}} \begin{array}{*{20}c} , & {i = 1,2,...,m} \\ \end{array}$$

Y-axis coordinate sequence:

11 $$\left( {y_{1} ,y_{2} ,...,y_{n} } \right)\begin{array}{*{20}c} , & {} \\ \end{array} y_{j} = y_{\min } + \left( {j - 1} \right) \cdot d_{{{\mathrm{grid}}}} \begin{array}{*{20}c} , & {j = 1,2,...,n} \\ \end{array}$$

where *m* is the number of grid points in the X direction, and *n* is the number of grid points in the Y direction, which are jointly determined by the size of the computational domain and the step size:

12 $$m = \frac{{x_{\max } - x_{\min } }}{{d_{{{\mathrm{grid}}}} }} + 1\begin{array}{*{20}c} , & {} \\ \end{array} n = \frac{{y_{\max } - y_{\min } }}{{d_{{{\mathrm{grid}}}} }} + 1$$

A regular particle set is generated by doubly traversing the above coordinate sequences. For each pair of coordinates (*x*_*i*_, *y*_*j*_), a particle Pij is created with its spatial coordinates ((*x*_*i*_, *y*_*j*_, 0). All particles are numbered sequentially in the order of X first and then Y, forming an ordered initial particle set:

13 $$G = \left\{ {P_{k} \left| {k = 1,2,...,N_{{{\mathrm{total}}}} } \right.} \right\}$$

where the total number of particles N_total_ = *m* × *n*, which is jointly determined by the size of the computational domain and the generation step size. The number *k* of each particle P_k_ and the grid indices (*i*, *j*) satisfy the following relationship:

14 $$k = \left( {j - 1} \right) \cdot m + i$$

### Random polygonal aggregate generation method

To construct a random aggregate distribution within the computational domain that conforms to engineering practice, this section proposes a controlled random iterative algorithm for generating a set of random polygonal aggregates that satisfy preset geometric constraints and spatial distribution constraints, as shown in Fig. 3b. Each aggregate A_k_(*k* = 1, 2, …, N_agg_) is defined by its vertex coordinate sequence:

15 $$V_{k} = \left[ {\left( {x_{1} ,y_{1} } \right),\left( {x_{2} ,y_{2} } \right),...,\left( {x_{{n_{s} }} ,y_{{n_{s} }} } \right)} \right]$$

where *n*_*s*_ is the number of edges of the polygon, and N_agg_ is the total number of target aggregates. The core of the algorithm lies in iterative generation and double verification, ensuring that each aggregate is completely within the computational domain and does not intersect with previously generated aggregates. The algorithm procedure is described as follows.

#### Random generation of aggregate geometric parameters

To improve the packing efficiency of aggregate distribution, a "large first, then small" generation strategy is adopted. First, N_agg_ random radii *r*_*k*_ are generated as follows:

16 $$r_{k} = R_{\min } + \left( {R_{\max } - R_{\min } } \right) \cdot {\mathrm{rand}}\begin{array}{*{20}c} , & {} \\ \end{array} k = 1,2,...,N_{agg}$$

where R_min_ and R_max_ are the user-defined minimum and maximum characteristic radii, respectively, and rand is a random number uniformly distributed in the interval [0, 1). The generated radius sequence is sorted in descending order to ensure that larger aggregates are placed first.

For the *k*-*th* candidate aggregate, the following basic geometric parameters are generated in sequence:

1. Characteristic radius *r*_*k*_: The pre-generated radius value is adopted as the circumscribed circle radius of the aggregate.

2. Center coordinates (*x*_*k*_, *y*_*k*_): To ensure that the entire aggregate is located within the computational domain, the sampling range of the center is constrained as follows:

17 $$x_{k} \in \left[ {x_{\min } + r_{k} \cdot \eta \begin{array}{*{20}c} , & {x_{\max } - r_{k} \cdot \eta } \\ \end{array} } \right]\begin{array}{*{20}c} , & {y_{k} \in \left[ {y_{\min } + r_{k} \cdot \eta \begin{array}{*{20}c} , & {y_{\max } - r_{k} \cdot \eta } \\ \end{array} } \right]} \\ \end{array}$$

where *η* is the boundary margin coefficient, which can be set by the user but should not be less than 0.5 to ensure that the aggregate does not touch the boundary. Uniform random sampling is performed within this rectangular region:

18 $$\begin{gathered} x_{k} = x_{\min } + r_{k} \cdot \eta + \left( {x_{\max } - x_{\min } - 2r_{k} \cdot \eta } \right) \cdot {\mathrm{rand}} \hfill \\ y_{k} = y_{\min } + r_{k} \cdot \eta + \left( {y_{\max } - y_{\min } - 2r_{k} \cdot \eta } \right) \cdot {\mathrm{rand}} \hfill \\ \end{gathered}$$

3. Number of edges *n*_*s*_: An integer is randomly generated within the user-defined interval:

19 $$n_{s} = {\mathrm{randi}}\left( {N_{\min } ,N_{\max } } \right)$$

where N_min_ and N_max_ are the minimum and maximum numbers of edges, respectively, and randi is a function that generates uniformly distributed random integers.

4. Irregularity *i*_*r*_: Controls the non-uniformity of the angular distribution of vertices, with a value range of [0,1]. The larger the *i*_*r*_, the more irregular the shape of the polygon.

5. Sharpness *s*_*p*_: Controls the degree of abrupt change in vertex radius, with a value range of [0,1]. The larger the *s*_*p*_, the higher the probability that the polygon will have sharp protrusions.

#### Generation of polygon vertex coordinates

Based on the above parameters, the polar coordinate method is adopted to generate the polygon vertices. First, *n*_*s*_ equally spaced angles are generated within the range of [0, 2π):

20 $$\theta_{i} = \frac{{2\pi \left( {i - 1} \right)}}{{n_{s} }}\begin{array}{*{20}c} , & {} \\ \end{array} i = 1,2,...,n_{s}$$

where *θ*_*i*_ is the initial polar angle of the *i*-*th* vertex.

Angle perturbation: A random perturbation is added to each base angle *θ*_*i*_:

21 $$\theta_{i}{^\prime} = \theta_{i} + i_{r} \cdot \varepsilon_{i} \cdot \frac{\pi }{{n_{s} }}\begin{array}{*{20}c} , & {} \\ \end{array} \varepsilon_{i} \sim N\left( {0,1} \right)$$

where *ε*_*i*_ are random numbers following the standard normal distribution, and N(0, 1) denotes the normal distribution with a mean of 0 and a variance of 1. The perturbed angles are sorted to ensure that the polygon vertices are arranged in counterclockwise order:

22 $$\theta_{\left( 1 \right)}{^\prime} \le \theta_{\left( 2 \right)}{^\prime} \le ... \le \theta_{{\left( {n_{s} } \right)}}{\prime}$$

Radius perturbation: A base radius vector *r*_*i*_ is generated, with its mean equal to the characteristic radius *r*_*k*_:

23 $$r_{i} = r_{k} \cdot \left( {0.8 + 0.4\xi_{i} } \right)\begin{array}{*{20}c} , & {} \\ \end{array} \xi_{i} \sim U\left( {0,1} \right)$$

where *ξ*_*i*_ are random numbers uniformly distributed in [0, 1).

Sharp features are introduced to some vertices:

24 $${\mathrm{if}}\begin{array}{*{20}c} {} & {\varsigma_{i} < s_{p} } \\ \end{array} \begin{array}{*{20}c} , & {\varsigma_{i} \sim U\left( {0,1} \right)} \\ \end{array} \begin{array}{*{20}c} , & {} \\ \end{array} \begin{array}{*{20}c} {{\mathrm{then}}} & {r_{i} = } \\ \end{array} r_{i} \cdot \left( {1.2 + 0.3\eta_{i} } \right)\begin{array}{*{20}c} , & {\eta_{i} \sim U\left( {0,1} \right)} \\ \end{array}$$

where *ζ*_*i*_ is a random number that determines whether to apply the sharp feature, and *η*_*i*_ is a sharpness control parameter.

To prevent excessive abrupt changes in radii between adjacent vertices, the radius sequence is smoothed. The smoothing function is defined as:

25 $$r_{i}^{{{\mathrm{smooth}}}} = \frac{1}{2w + 1}\sum\limits_{j = i - w}^{i + w} {r_{{\bmod \left( {j,n_{s} } \right)}} } \begin{array}{*{20}c} , & {} \\ \end{array} i = 1,2,...,n_{s}$$

where *w* is the smoothing window radius, taken as *w* = 1 in this paper; mod(*j*, *n*_*s*_) is the modulo operation, ensuring correct values at the cyclic boundary.

The final polygon vertex coordinates are determined by the following equation:

26 $$\begin{gathered} x_{i} = x_{k} + r_{i}^{{{\mathrm{smooth}}}} \cdot \cos \left( {\theta_{i}{^\prime} } \right) \hfill \\ y_{i} = y_{k} + r_{i}^{{{\mathrm{smooth}}}} \cdot \sin \left( {\theta_{i}{^\prime} } \right) \hfill \\ \end{gathered}$$

The first and last vertices are connected to form a closed polygon:

27 $$V_{k} = \left[ {\left( {x_{1} ,y_{1} } \right),\left( {x_{2} ,y_{2} } \right),...,\left( {x_{{n_{s} }} ,y_{{n_{s} }} } \right),\left( {x_{1} ,y_{1} } \right)} \right]$$

#### Spatial constraint verification

The generated candidate aggregate must pass double verification to be accepted:

**Boundary verification: Compute the bounding box of the polygon:**

28 $$\begin{gathered} x_{\min }^{\left( k \right)} = \mathop {\min }\limits_{i = 1}^{{n_{s} }} x_{i} \begin{array}{*{20}c} , & {} \\ \end{array} x_{\max }^{\left( k \right)} = \mathop {\max }\limits_{i = 1}^{{n_{s} }} x_{i} \hfill \\ y_{\min }^{\left( k \right)} = \mathop {\min }\limits_{i = 1}^{{n_{s} }} y_{i} \begin{array}{*{20}c} , & {} \\ \end{array} y_{\max }^{\left( k \right)} = \mathop {\max }\limits_{i = 1}^{{n_{s} }} y_{i} \hfill \\ \end{gathered}$$

Verify whether the bounding box is completely within the computational domain:

29 $$\begin{gathered} x_{\min }^{\left( k \right)} \ge x_{{{\mathrm{min}}}} \begin{array}{*{20}c} , & {} \\ \end{array} x_{\max }^{\left( k \right)} \le x_{\max } \hfill \\ y_{\min }^{\left( k \right)} \ge y_{{{\mathrm{min}}}} \begin{array}{*{20}c} , & {} \\ \end{array} y_{\max }^{\left( k \right)} \le y_{\max } \hfill \\ \end{gathered}$$

Overlap verification: To prevent aggregates from overlapping or intersecting with each other in space, the spatial relationship between the current candidate aggregate and all previously generated aggregates must be verified. To improve computational efficiency, a two-stage verification strategy is adopted:

1. Fast bounding box exclusion: For the bounding box B_k_ of the current aggregate and the bounding box B_j_ of a previously generated aggregate A_j_, if B_k_ and B_j_ do not intersect, the aggregate can be directly skipped without the need for precise determination. The condition for bounding box intersection is:

30 $$\begin{gathered} x_{\min }^{\left( k \right)} \le x_{{{\mathrm{max}}}}^{\left( j \right)} \begin{array}{*{20}c} {} & {{\mathrm{and}}} & {} \\ \end{array} x_{\max }^{\left( k \right)} \ge x_{\min }^{\left( j \right)} \hfill \\ y_{\min }^{\left( k \right)} \le y_{{{\mathrm{max}}}}^{\left( j \right)} \begin{array}{*{20}c} {} & {{\mathrm{and}}} & {} \\ \end{array} y_{\max }^{\left( k \right)} \ge y_{\min }^{\left( j \right)} \hfill \\ \end{gathered}$$

2. Precise polygon intersection determination: If the bounding boxes intersect, the Separating Axis Theorem (SAT) is adopted for precise intersection detection. The core of this theorem is: if two convex polygons do not intersect, there exists a separating axis such that the projections of the two polygons onto that axis do not overlap.

For polygons P and Q, the SAT algorithm checks all possible separating axes: the normal vectors of each edge of P and the normal vectors of each edge of Q.

For an edge E = *V*_*i*+1_—*V*_*i*_ of a polygon, its normal vector *n* is calculated as:

31 $$n = \frac{{\left( { - E_{y} ,E_{x} } \right)}}{\left\| E \right\|}$$

For each candidate axis *n*, the projection intervals of the two polygons onto that axis are calculated:

32 $$\begin{gathered} {\mathrm{proj}}_{P} = \left[ {\mathop {\min }\limits_{i} \left( {p_{i} \cdot n} \right)\begin{array}{*{20}c} , & {\mathop {\max }\limits_{i} \left( {p_{i} \cdot n} \right)} \\ \end{array} } \right] \hfill \\ {\mathrm{proj}}_{Q} = \left[ {\mathop {\min }\limits_{j} \left( {q_{i} \cdot n} \right)\begin{array}{*{20}c} , & {\mathop {\max }\limits_{j} \left( {q_{j} \cdot n} \right)} \\ \end{array} } \right] \hfill \\ \end{gathered}$$

where *p*_*i*_ and *q*_*j*_ are the vertex vectors of polygons P and Q, respectively.

If there exists an axis such that the projection intervals do not overlap, i.e.:

33 $${\mathrm{max}}\left( {{\mathrm{proj}}_{P} } \right) < \min \left( {{\mathrm{proj}}_{Q} } \right)\begin{array}{*{20}c} {} & {{\mathrm{or}}} & {{\mathrm{max}}\left( {{\mathrm{proj}}_{Q} } \right) < \min \left( {{\mathrm{proj}}_{P} } \right)} \\ \end{array}$$

Then the two polygons are determined to be non-intersecting; otherwise, they intersect.

#### Aggregate acceptance and iterative loop

The verification result determines whether a candidate aggregate is accepted:

If it passes the boundary verification and does not intersect with any previously generated aggregates, then the aggregate is accepted and added to the aggregate set A:

34 $$A = A \cup \left\{ {A_{k} } \right\}$$

If the verification fails, the aggregate is rejected, and the process returns to “Random generation of aggregate geometric parameters” section to regenerate a set of random parameters.

The system iteratively executes the above steps until the specified number N_agg_ of aggregates is successfully generated, or until a preset maximum number of attempts T_max_ is reached:

35 $$T_{{{\mathrm{total}}}} \le T_{{{\mathrm{max}}}} \begin{array}{*{20}c} {} & {{\mathrm{and}}} & {} \\ \end{array} \left| A \right| = N_{{{\mathrm{agg}}}}$$

where T_total_ is the total number of attempts, and |A| is the number of aggregates that have been generated.

### Generation of the particle computational model for random polygonal aggregates

Based on the previously generated random polygonal aggregate set, this step achieves automatic classification of each particle through precise geometric inclusion determination, ultimately constructing a "particle computational model for random polygonal aggregates" that can be used for numerical simulation, as shown in Fig. 3c. The core of this model is to assign a clear material property label to each particle, with the classification based on the spatial geometric relationship between the particle and the polygonal aggregates.

#### Fast exclusion and precise determination strategy

To improve computational efficiency, a two-stage determination strategy is also adopted:

**Stage 1: Bounding box fast exclusion**

For particle *P*_*i*_(*x*_*p*_, *y*_*p*_) and aggregate A_k_, first check whether the particle is located within the bounding box of the aggregate:

36 $${\mathrm{if}}\,x_{\min }^{\left( k \right)} \begin{array}{*{20}c} < & {{\mathrm{or}}} & {} \\ \end{array} x_{p} > x_{{{\mathrm{max}}}}^{\left( k \right)} \begin{array}{*{20}c} {} & {{\mathrm{or}}} & {} \\ \end{array} y_{p} < y_{\min }^{\left( k \right)} \begin{array}{*{20}c} {} & {{\mathrm{or}}} & {} \\ \end{array} y_{p} > y_{{{\mathrm{max}}}}^{\left( k \right)}$$

Then particle P_i_ is definitely outside the aggregate and can be directly excluded without the need for precise determination.

**Stage 2: Precise determination using the ray casting method**

For particles that pass the bounding box check, the ray casting method is adopted to determine whether the particle is inside the polygon. The basic principle is: a ray is cast from the target point along the positive X direction, and the number of intersections between this ray and the polygon edges is counted. If the number of intersections is odd, the point is inside the polygon; if even, it is outside.

For an edge *V*_*i*_*V*_*i*+1_ of the polygon, the condition for the ray from point P(*x*_*p*_, *y*_*p*_) to intersect with the edge is:

37 $$\left( {y_{p} > y_{i} } \right) \ne \left( {y_{p} > y_{i + 1} } \right)\begin{array}{*{20}c} {} & {{\mathrm{and}}} & {} \\ \end{array} x_{p} < \frac{{\left( {x_{i + 1} - x_{i} } \right)\left( {y_{p} - y_{i} } \right)}}{{y_{i + 1} - y_{i} }} + x_{i}$$

where the first condition ensures that the ray passes through the horizontal interval of the edge, and the second condition calculates the X-coordinate of the intersection point and ensures that the ray intersects the edge.

The intersection counting function cross(P, V) is defined as:

38 $${\mathrm{cross}}\left( {P,V} \right) = \sum\limits_{i = 1}^{{n_{s} }} {1_{{{\mathrm{intersect}}}} \left( {P,V_{i} V_{i + 1} } \right)}$$

where 1_intersect_ is an indicator function that takes the value 1 when the ray intersects the edge, and 0 otherwise.

The inclusion relationship between point P and polygon V is determined as:

39 $${\mathrm{inside}}\left( {P,V} \right) = \left\{ {\begin{array}{*{20}l} {{\mathrm{true}}} \hfill & {\begin{array}{*{20}l} {} \hfill & {{\mathrm{cross}}\left( {P,V} \right)} \hfill & {{\mathrm{is}}\,{\mathrm{odd}}\,{\mathrm{number}}} \hfill \\ \end{array} } \hfill \\ {{\mathrm{false}}} \hfill & {\begin{array}{*{20}c} {} & {{\mathrm{cross}}\left( {P,V} \right)} & {{\mathrm{is}}\,{\mathrm{even}}\,{\mathrm{number}}} \\ \end{array} } \hfill \\ \end{array} } \right.$$

#### Calculation of the minimum distance from a particle to the aggregate network

For a particle *P*_*i*_, the minimum distance *d*_min_ from it to the entire aggregate network is obtained by traversing all aggregates and calculating the minimum distance from the point to each polygon:

40 $$d_{\min } \left( {P_{i} } \right) = \mathop {\min }\limits_{k = 1}^{{N_{agg} }} d\left( {P_{i} ,A_{k} } \right)$$

where d(*P*_*i*_, *A*_*k*_) represents the minimum Euclidean distance from point *P*_*i*_ to polygon *A*_*k*_. For a polygon, the minimum distance from a point to the polygon is defined as the minimum of the distances from the point to all edges of the polygon:

41 $$d\left( {P,A_{k} } \right) = \mathop {\min }\limits_{i = 1}^{{n_{s} }} d\left( {P,E_{i}^{\left( k \right)} } \right)$$

where *E(k) i* is the *i*-*th* edge of polygon *A*_*k*_, defined by vertices *V*_*i*_ and *V*_*i*+1_.

For a line segment AB defined by endpoints A(*x*_1_, *y*_1_) and B(*x*_2_, *y*_2_), the minimum distance from point P(*x*_*p*_, *y*_*p*_) to the line segment is calculated as:

42 $$\begin{gathered} t = \frac{{\left( {x_{p} - x_{1} } \right)\left( {x_{2} - x_{1} } \right) + \left( {y_{p} - y_{1} } \right)\left( {y_{2} - y_{1} } \right)}}{{\left( {x_{2} - x_{1} } \right)^{2} + \left( {y_{2} - y_{1} } \right)^{2} }} \\ d\left( {P,AB} \right) = \left\{ {\begin{array}{*{20}l} {\sqrt {\left( {x_{p} - x_{1} } \right)^{2} + \left( {y_{p} - y_{1} } \right)^{2} } } \hfill & {t \le 0} \hfill \\ {\sqrt {\left( {x_{p} - x_{2} } \right)^{2} + \left( {y_{p} - y_{2} } \right)^{2} } } \hfill & {t \ge 1} \hfill \\ {\sqrt {\left( {x_{p} - \left[ {x_{1} + t\left( {x_{2} - x_{1} } \right)} \right]} \right)^{2} + \left( {y_{p} - \left[ {y_{1} + t\left( {y_{2} - y_{1} } \right)} \right]} \right)^{2} } } \hfill & {0 < t < 1} \hfill \\ \end{array} } \right. \\ \end{gathered}$$

where the parameter t represents the projection position of point P onto the line containing segment AB. *t* ≤ 0 indicates that the projection point lies outside the segment beyond endpoint A, *t* ≥ 1 indicates that the projection point lies outside the segment beyond endpoint B, and 0 < *t* < 1 indicates that the projection point lies inside the segment.

#### Particle attribute classification and model generation

All particles are traversed, and the above determination is performed for each particle *P*_*i*_: initialize inside = false, aggID = 0, and for each aggregate A_k_ ∈ A, if xp is within the bounding box of the aggregate, then determine whether point P_i_ is inside polygon *V*_*k*_:

43 $${\mathrm{inside}}_{k} = {\mathrm{inside}}\left( {P_{i} ,V_{k} } \right)$$

If insidek = true, then:

44 $${\mathrm{inside}} = {\mathrm{true}}\begin{array}{*{20}c} , & {{\mathrm{aggID}}} \\ \end{array} = k\begin{array}{*{20}c} , & {\text{break out of the loop}} \\ \end{array}$$

The binary classification of particle attributes is completed based on the determination result:

If inside = true: particle P_i_ is classified as an “aggregate region particle”. Particles in this region represent the aggregate material and should be assigned material parameters reflecting the mechanical properties of the aggregate in subsequent numerical simulations.

If inside = false: particle P_i_ is classified as a “matrix region particle”. Particles in this region represent the matrix material surrounding the aggregates.

The classification function Φ(P_i_) is defined as:

45 $$\Phi \left( {P_{i} } \right) = \left\{ {\begin{array}{*{20}l} 1 \hfill & {\text{aggregate region point}} \hfill \\ 0 \hfill & {\text{matrix region point}} \hfill \\ \end{array} } \right.$$

After completing the calculation and classification for all particles, the system automatically generates a structured particle computational model:

46 $$M = \left\{ {\begin{array}{*{20}c} {\left( {P_{i} ,\Phi \left( {P_{i} } \right),{\mathrm{aggID}}_{i} } \right)} & {|} & {i = 1,2,...,N_{{{\mathrm{total}}}} } \\ \end{array} } \right\}$$

This model records the spatial coordinates and attribute labels (“aggregate region” or “matrix region”) of each particle, and outputs structured data files containing information such as particle number, coordinates, and classification identifier. These data files can be directly used for assigning differentiated material parameters and performing computational analyses in subsequent numerical simulations.

## Numerical example analysis of concrete with irregular Aggregates

**Fig. 4:**
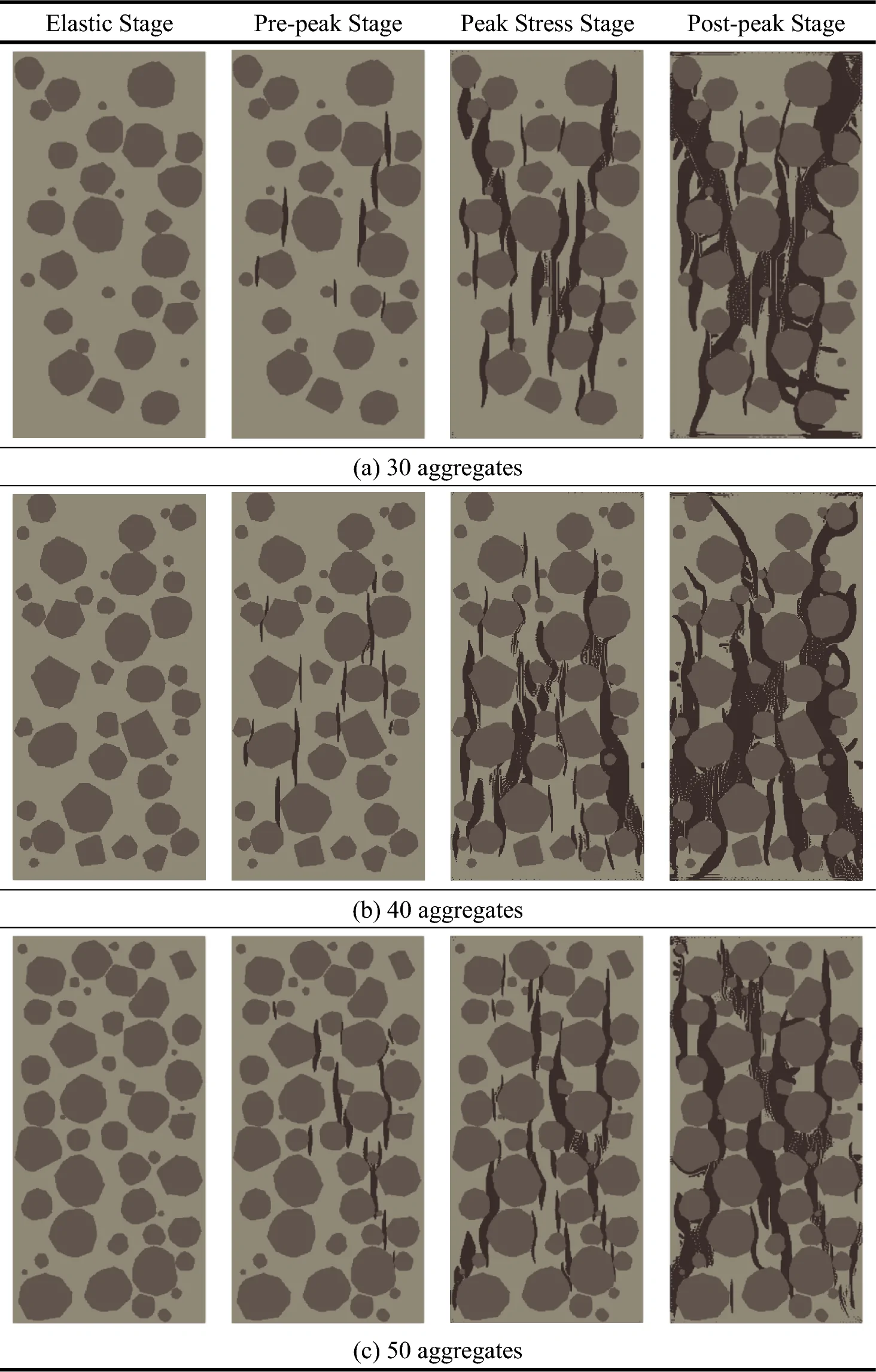
Crack evolution morphology of concrete under uniaxial compression at different aggregate densities (from left to right: Elastic Stage, Pre-peak Stage, Peak Stress Stage, Post-peak Stage): (**a**) 30 aggregates; (**b**) 40 aggregates; (**c**) 50 aggregates.

**Fig. 5:**
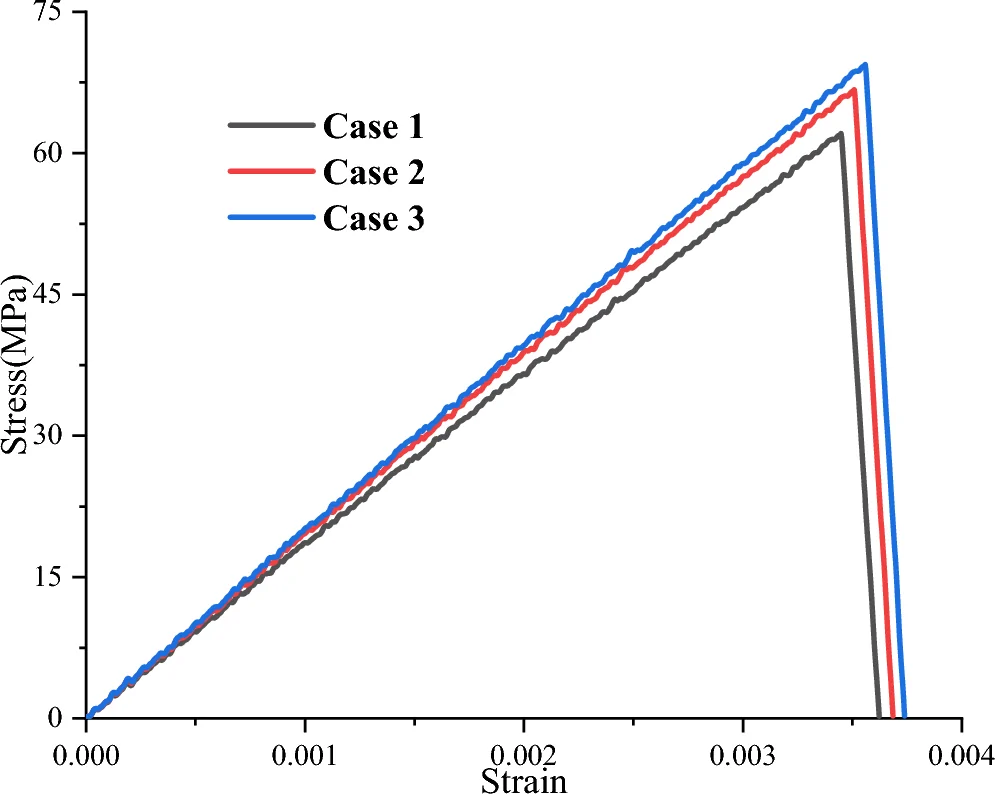
Uniaxial compressive stress–strain curves for different aggregate densities.

**Fig. 6:**
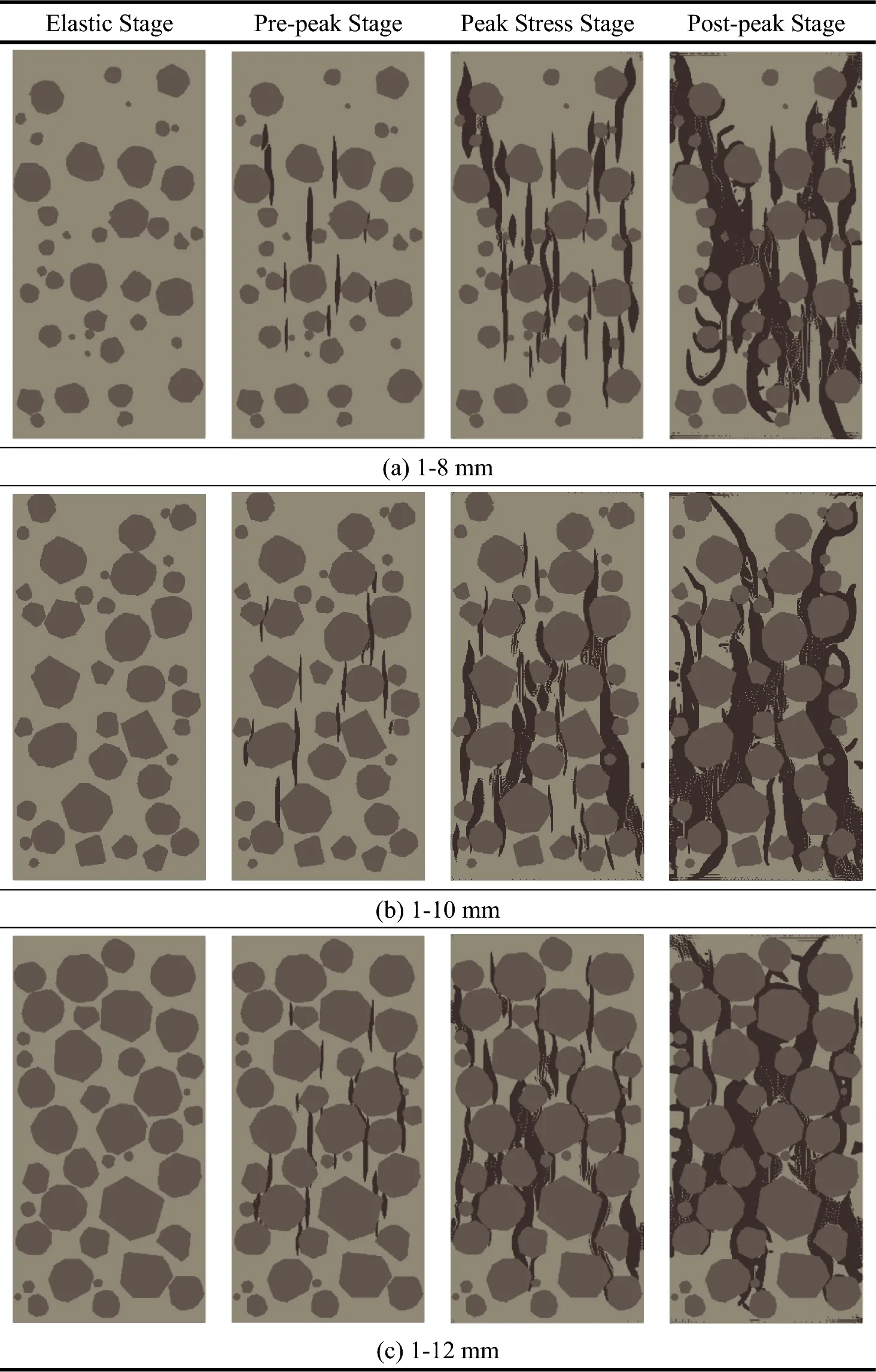
Crack evolution morphology of concrete under uniaxial compression under different aggregate size ranges: (**a**) 1–8 mm; (**b**) 1–10 mm; (**c**) 1–12 mm.

In this study, random aggregate models are generated based on the MATLAB platform, and numerical simulations are conducted within a two-dimensional rectangular computational domain of 100 mm × 200 mm (i.e., 0.1 m × 0.2 m). A grid step size of 0.5 mm is adopted for discretization, generating a total of 78,804 regularly arranged initial particles. Irregular aggregates are randomly distributed within the computational domain to construct a numerical model of the concrete mesostructure, thereby simulating the mesoscopic compositional characteristics of real concrete. The material parameters for the aggregate and matrix phases are adopted from Peng et al. (2019) which also employs a two-dimensional mesoscale model to investigate concrete compression failure, providing good comparability with the present study. The aggregate density is 2700 kg/m^3^ with a Poisson’s ratio of 0.16, and the matrix density is 2100 kg/m^3^ with a Poisson’s ratio of 0.22. All other mechanical parameters are consistent with those in Ref.^10^. Since this study focuses on the relative effects of aggregate geometric characteristics rather than the precise prediction of absolute strength, the literature parameters are directly adopted without additional calibration, and all cases use identical parameters to ensure the validity of the comparative results. It should be noted that the failure evolution process for each numerical example in Figs. 4, 5 and 6 is presented at four typical loading stages, namely: Elastic Stage, Pre-peak Stage, Peak Stress Stage, and Post-peak Stage.

To investigate the influence of irregular aggregate geometric characteristics on the macroscopic mechanical behavior of concrete, three sets of comparative numerical examples are designed using the control variable method, as shown in Table 1. The first set (Cases 1–3) fixes the size range at 1–10 mm and the edge number range at 8–20, while varying the number of aggregates (30, 40, 50) to study the effect of aggregate density. The second set (Cases 4–6) fixes the number of aggregates at 40 and the edge number range at 8–20, while varying the size range (1–8 mm, 1–10 mm, 1–12 mm) to study the effect of size distribution. The third set (Cases 7–9) fixes the number of aggregates at 40 and the size range at 1–10 mm, while varying the edge number range (8–15, 8–20, 8–25) to study the effect of shape complexity.

**Table 1:** Design table of aggregate geometric characteristic parameters.

Group	Case	Number of aggregates	Aggregate size range (mm)	Aggregate edge number range	Aggregate volume fraction (%)
Group 1	1	30	1–10	8–20	32.78
	2	40			38.06
	3	50			48.84
Group 2	4	40	1–8	8–20	23.75
	5		1–10		38.06
	6		1–12		52.20
Group 3	7	40	1–10	8–15	44.71
	8			8–20	38.06
	9			8–25	45.67

The randomly generated aggregate models described above are imported into the SPH program to conduct uniaxial compression numerical simulations. By analyzing the failure morphology, stress–strain curves, peak strength, peak strain, and other characteristics during the tests, the regulatory mechanism of irregular aggregate geometric parameters on the macroscopic mechanical behavior of concrete is revealed. The simulation results of the three sets of comparative examples are analyzed and discussed below.

### Number of Aggregates: 30, 40, 50

With the aggregate size range fixed at 1–10 mm and the edge number range fixed at 8–20, three cases with aggregate numbers of 30, 40, and 50 are considered. The detailed geometric parameters and volume fractions are provided in Table 1. This subsection first compares the meso-crack evolution and failure modes under different aggregate densities (Fig. 4), and then combines the macroscopic stress–strain curves with quantitative indicators such as peak stress and peak strain (Fig. 5) to systematically reveal the regulatory effect of aggregate density on the meso-damage and macroscopic mechanical performance of concrete.

Aggregate density has a significant influence on the meso-damage evolution and macroscopic failure mode of concrete (Fig. 4). As aggregate density increases, the crack morphology exhibits notable changes. As shown in Fig. 4a (30 aggregates, low density), the aggregate distribution is sparse with large inter-particle spacing. Stress concentrates at individual aggregate corners and interfaces, and cracks propagate along a few dominant paths, forming a localized vertical shear failure band with strong connectivity. The matrix bears most of the load, and damage is primarily characterized by localized concentrated shear. As shown in Fig. 4b (40 aggregates, medium density), the aggregate distribution is uniform with moderate inter-particle spacing. The superposition of stress fields between aggregates increases the number of microcrack initiation sites, and the propagation paths interfere with each other, forming diffuse shear failure with a wider damaged region. The synergistic load-bearing interaction between aggregates and matrix is enhanced. As shown in Fig. 4c (50 aggregates, high density), the aggregate distribution is dense with extremely small inter-particle gaps. The proportion of corners and interfaces increases significantly, and stress concentration becomes globally distributed. Microcracks initiate rapidly along interfaces and corners and interconnect with each other, forming a corner-interface-dominated diffuse failure zone that covers the entire specimen, with more pronounced brittle characteristics.

Figure 5 presents the complete uniaxial compressive stress–strain relationships for the three cases. All three curves exhibit three typical stages: linear elastic rise, peak strengthening, and post-peak decline. As the number of aggregates increases, the peak stress, peak strain, and post-peak characteristics show consistent trends. The mechanical parameters for the three cases are as follows: for 30 aggregates, peak stress = 62.18 MPa, peak strain = 0.00345; for 40 aggregates, peak stress = 66.85 MPa, peak strain = 0.00351; for 50 aggregates, peak stress = 69.52 MPa, peak strain = 0.00356. From these data and Fig. 5, it can be seen that as the number of aggregates increases from 30 to 50, the peak compressive strength gradually increases from 62.18 MPa to 69.52 MPa, while the peak strain increases slightly from 0.00345 to 0.00356. It should be noted that the binary fracture model adopted in this study does not consider progressive damage evolution prior to peak stress. Therefore, the simulated stress–strain curves are characterized by linear growth followed by an abrupt drop after the peak, which differs from the nonlinear hardening and gradual softening behaviour of real concrete under compression. Nevertheless, all numerical cases share the same model settings, so the relative differences in stress–strain responses among different aggregate parameters are reliable for comparative analysis.

To further quantitatively analyze crack propagation, the crack area ratio (the ratio of failed particles to total particles) at the peak stress stage was calculated for each case. The results show that for 30 aggregates, the crack area ratio is only 7.2%, and cracks are primarily localized. For 40 aggregates, the crack area ratio increases to 10.5%, and the damage distribution begins to transition toward a more diffuse pattern. For 50 aggregates, the crack area ratio reaches 14.8%, and the damage exhibits a fully diffuse distribution across the entire specimen.

These quantitative results demonstrate that increasing aggregate density effectively enhances the ultimate load-bearing capacity and peak deformation capacity of the specimen. Moreover, higher aggregate density is accompanied by more pronounced brittle characteristics and more diffuse damage distribution.

### Aggregate size ranges: 1–8 mm, 1–10 mm, 1–12 mm

The number of aggregates is fixed at 40 and the edge number range at 8–20, while the size range is varied among 1–8 mm, 1–10 mm, and 1–12 mm to investigate the influence of size distribution on the failure mode and mechanical performance of concrete. The crack evolution morphologies are shown in Fig. 6.

As the size range expands, the crack morphology exhibits significant changes (Fig. 6). As shown in Fig. 6a (1–8 mm, small size range), the aggregates are predominantly small-sized with uniform distribution and a moderate interface proportion. Cracks propagate along a few dominant paths, forming a concentrated vertical shear failure band with strong connectivity, and the matrix bears most of the load. As shown in Fig. 6b (1–10 mm, medium size range), large and small aggregates are interleaved with a moderate size gradation. The number of microcrack initiation sites increases, and the propagation paths interfere with each other, exhibiting diffuse shear failure characteristics with a wider damaged region. As shown in Fig. 6c (1–12 mm, large size range), the proportion of large aggregates is high, and the local spacing is reduced. Stress concentration at the corners of large aggregates is significantly enhanced, and cracks rapidly propagate and connect along the edges of large aggregates, forming a large-aggregate-corner-dominated diffuse failure zone with prominent brittle characteristics.

**Fig. 7:**
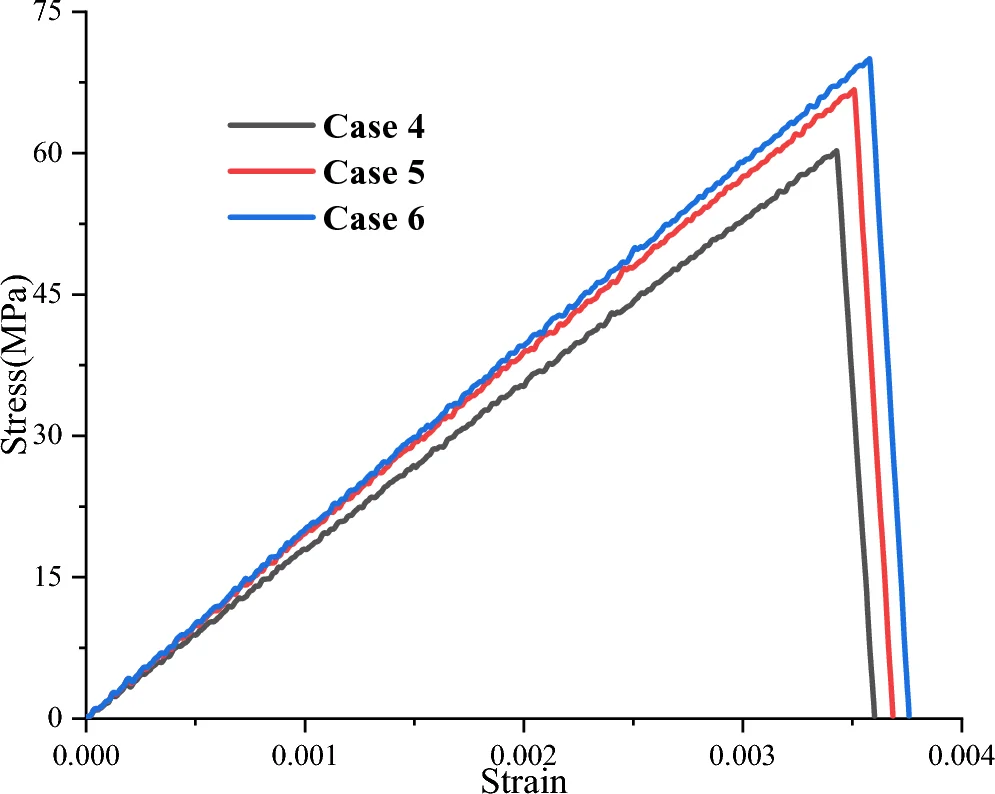
Uniaxial compressive stress–strain curves for different aggregate size ranges.

Figure 7 presents the stress–strain curves for the three cases. The corresponding mechanical parameters are as follows: for the 1–8 mm range, peak stress = 60.35 MPa, peak strain = 0.00343; for the 1–10 mm range, peak stress = 66.85 MPa, peak strain = 0.00351; for the 1–12 mm range, peak stress = 70.14 MPa, peak strain = 0.00358. As the size range expands, both peak strength and peak strain increase slightly.

Further statistical analysis of the crack area ratio at the peak stage shows values of 4.8% for the 1–8 mm range, 8.5% for the 1–10 mm range, and a significant increase to 13.2% for the 1–12 mm range, exhibiting a substantial upward trend. These quantitative results indicate that expanding the size range increases the proportion of large aggregates and the volume fraction, thereby enhancing the peak strength while promoting more diffuse crack propagation.

### Aggregate edge number ranges: 8–15, 8–20, 8–25

**Fig. 8:**
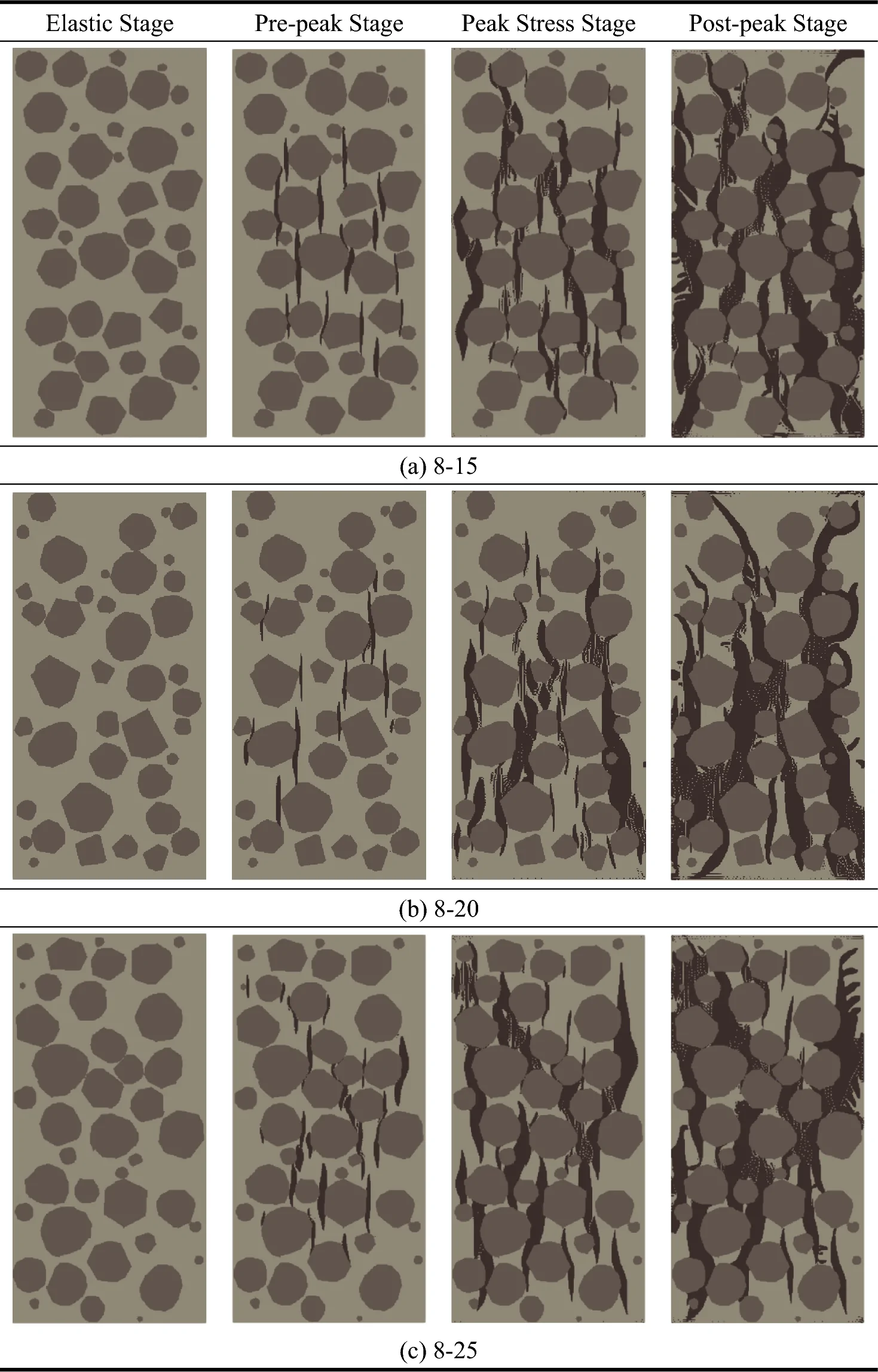
Crack propagation patterns of concrete under uniaxial compression with different aggregate shape complexities: (**a**) number of sides 8–15; (**b**) number of sides 8–20; (**c**) number of sides 8–25.

The number of aggregates is fixed at 40 and the size range at 1–10 mm, while the edge number range is varied among 8–15, 8–20, and 8–25 to investigate the influence of shape complexity on the failure mode and mechanical performance of concrete. The crack propagation morphologies are shown in Fig. 8.

As shape complexity increases, the crack morphology exhibits significant changes (Fig. 8). As shown in Fig. 8a (8–15 edges, low shape complexity), the aggregates have sharp corners, resulting in extremely strong stress concentration. Cracks initiate rapidly at a few sharp corners and propagate along preferential paths, forming a concentrated vertical shear failure band with strong connectivity and obvious brittle characteristics. As shown in Fig. 8b (8–20 edges, medium shape complexity), the distribution of corners and interfaces becomes more uniform. The number of microcrack initiation sites increases, and the propagation paths interfere with each other, forming a diffuse shear failure band with a wider damaged region and enhanced aggregate-matrix interaction. As shown in Fig. 8c (8–25 edges, high shape complexity), the aggregate profile approaches a circle with blunted corners, and stress concentration is significantly dispersed. Microcracks initiate uniformly along interfaces and interweave with each other, forming an interface-dominated fine diffuse failure band. The failure range is extensive, but the connectivity of individual cracks is weak, and the ductility of the material is relatively improved.

**Fig. 9:**
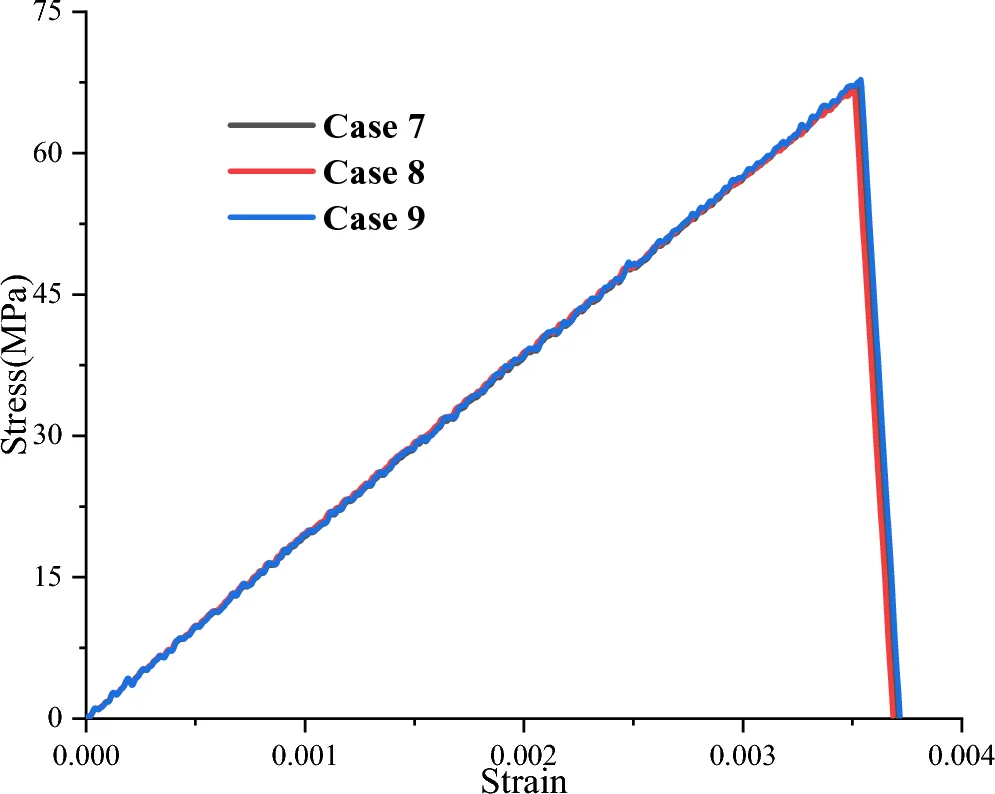
Uniaxial compressive stress–strain curves for different aggregate shape complexities.

Figure 9 presents the stress–strain curves for the three cases. The corresponding mechanical parameters are as follows: for 8–15 edges, peak stress = 67.43 MPa, peak strain = 0.00353; for 8–20 edges, peak stress = 66.85 MPa, peak strain = 0.00351; for 8–25 edges, peak stress = 67.98 MPa, peak strain = 0.00354. The volume fractions of the three cases are similar (ranging from 38.06% to 45.67%), and the differences in peak stress are small (approximately ± 1.6%). However, the post-peak curve morphologies differ markedly, indicating that increasing shape complexity helps improve material ductility.

Further statistical analysis of the crack area ratio at the peak stage shows values of 10.2% for 8–15 edges, 8.5% for 8–20 edges, and 11.5% for 8–25 edges. The ratios exhibit a pattern of being higher at both ends and lower in the middle. Notably, the crack area ratio for the smoother aggregates (8–25 edges) is slightly higher than that for the sharp-cornered aggregates (8–15 edges), indicating that smoother aggregates result in more uniform stress distribution and more fully developed cracking, which is consistent with the observed improvement in ductility.

## Comparison and verification with existing studies

**Fig. 10:**
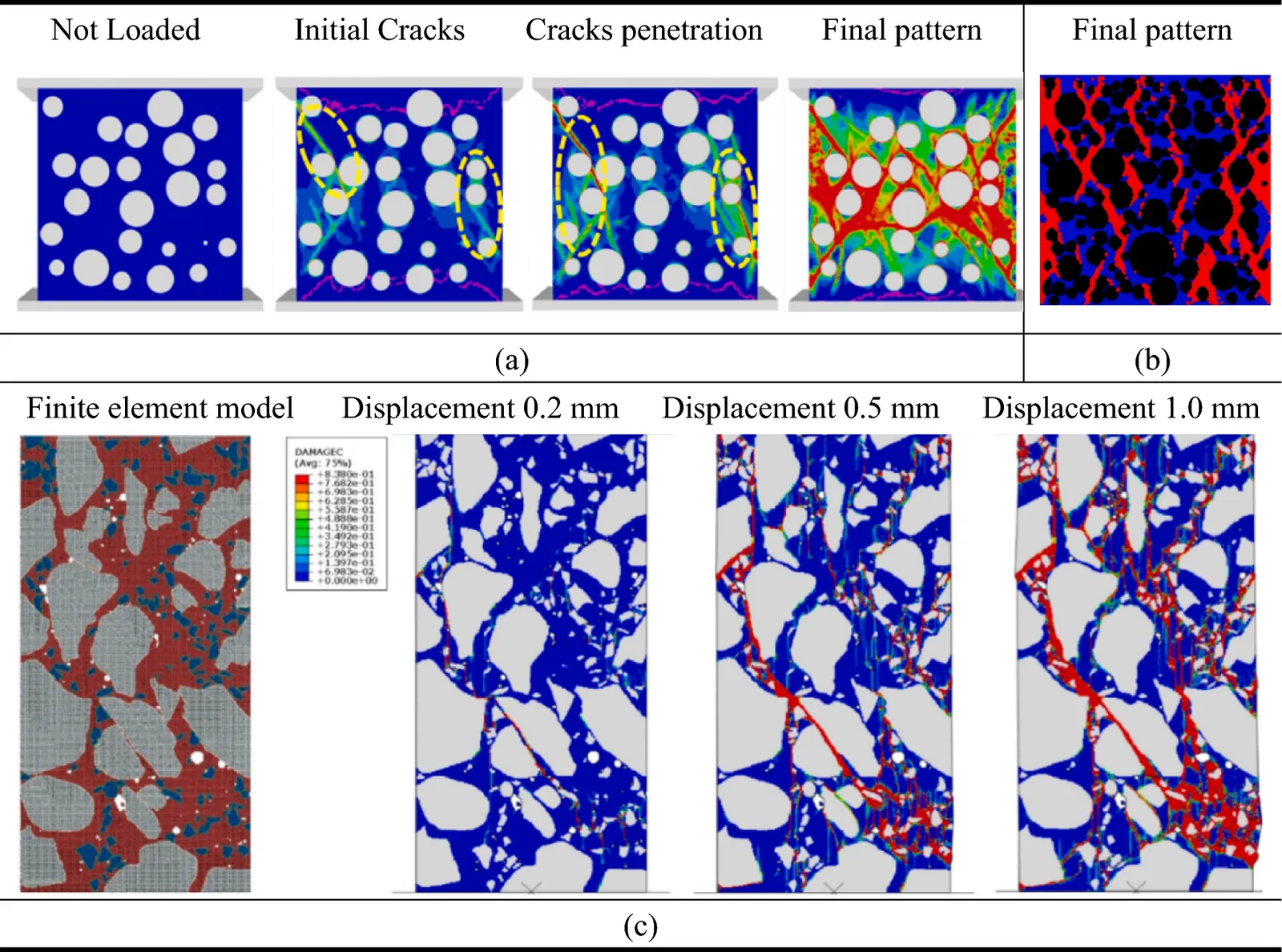
Comparison of crack evolution from reference literature: (**a**) circular aggregates with low filling rate (adapted from Ref.^11^); (**b**) circular aggregates with high filling rate (adapted from Ref.^14^); (**c**) polygonal aggregates (adapted from Ref.^13^).

This chapter selects classic mesoscale simulation results from the literature to benchmark the numerical examples in this study, as shown in Fig. 10. For the aggregate filling rate and size effects (“Number of aggregates: 30, 40, 50” and “Aggregate size ranges: 1–8 mm, 1–10 mm, 1–12 mm” sections), studies on circular aggregates in conventional concrete^11,14^ are adopted as the validation benchmark. For the aggregate shape complexity effect (“Aggregate edge number ranges: 8–15, 8–20, 8–25” section), a study on polygonal aggregates is adopted as the validation benchmark^13^. Figure 10a (circular aggregates with low filling rate, adapted from Ref.^11^) and Fig. 10b (circular aggregates with high filling rate, adapted from Ref.^14^) form a comparison of aggregate volume fraction from low to high. It should be noted that the variation in the number of aggregates in “Number of aggregates: 30, 40, 50” section and the variation in the size range in Section "Aggregate size ranges: 1–8 mm, 1–10 mm, 1–12 mm" both essentially manifest as differences in aggregate volume fraction. Therefore, the comparison of high and low filling rates in Fig. 10a and b can be jointly used to validate the core findings of these two sections. Figure 10c (polygonal aggregates, adapted from Ref.^13^) is used to validate the aggregate shape complexity effect in “Aggregate edge number ranges: 8–15, 8–20, 8–25” section. Although this study focuses on crumb rubber concrete, the controlling mechanism of polygonal aggregate morphology on crack initiation and propagation is consistent with that of conventional concrete. Moreover, this study established a high-precision real mesostructural model based on CT in-situ tests, providing significant validation value.

### Benchmark validation of aggregate filling rate and size effects

The meso-scale simulation results of circular aggregates from the literature (Fig. 10a low filling rate, Fig. 10b high filling rate) are shown below.

From the meso-crack evolution, it can be observed that in the low filling rate model (Fig. 10a), the aggregate distribution is sparse with large inter-particle spacing. Under compression, only a few dominant cracks are generated, which develop along a single preferential path, forming a localized concentrated shear failure band with limited damage scope and relatively low overall crack development. In contrast, in the high filling rate model (Fig. 10b), the aggregate distribution is dense with a significantly increased number of interfaces. Stress concentration points are distributed throughout the domain, and microcracks initiate simultaneously at multiple locations and interconnect with each other, ultimately transitioning from a single concentrated shear failure to a fully diffuse damage pattern, with substantially increased crack development range and connectivity.

The above findings from the literature are highly consistent with the conclusions of “Number of aggregates: 30, 40, 50” and “Aggregate size ranges: 1–8 mm, 1–10 mm, 1–12 mm” section of this study:

1. Filling rate and crack density: In this study, as the number of aggregates increases from 30 to 50 (i.e., increasing volume fraction) and as the size range expands from 1–8 mm to 1–12 mm (i.e., increasing overall filling rate), the crack area ratio at the peak stress stage increases significantly, and the failure mode similarly transitions from localized concentrated shear to diffuse damage. This trend is fully consistent with the crack evolution trends of circular aggregates under high and low filling rates in the literature.
2. Filling rate and brittle characteristics: In the literature, the high filling rate case exhibits faster post-peak crack propagation and a more abrupt stress drop, with more pronounced brittle characteristics. This corroborates the findings of this study: a larger number of aggregates, a wider size range, and a higher filling rate lead to greater post-peak brittleness and more diffuse damage. For further quantitative comparison, this study calculated the crack area ratio (ratio of failed particles to total particles) under different conditions. The results show that as the filling rate increases, the crack area ratio increases significantly from 4.8% (low filling rate) to 14.8% (high filling rate), which is fully consistent with the crack development trends under high and low filling rates in Refs.^11,14^. This quantitatively validates the accuracy of the proposed model in representing the effect of aggregate filling rate.
3. Size effect: In the literature, larger circular aggregates are more prone to inducing stress concentration at their corners and interfaces, accelerating crack propagation and connectivity. This supports the findings of “Aggregate size ranges: 1–8 mm, 1–10 mm, 1–12 mm” seciton of this study, where an increased proportion of large aggregates intensifies crack development and increases peak strength.

### Benchmark validation of aggregate shape complexity effects

The mesoscale simulation results for polygonal aggregates from the literature are shown in Fig. 10c. The results indicate that polygonal aggregates with sharp corners are prone to significant stress concentration at the corner locations. Microcracks preferentially initiate at the sharp corners and propagate rapidly along fixed paths, forming a highly connected concentrated shear failure surface with prominent brittle characteristics. In contrast, polygonal aggregates with a more rounded profile and a larger number of edges exhibit blunted corners and dispersed stress concentration. Cracks are no longer concentrated along a few dominant paths but instead initiate at multiple locations along the aggregate-matrix interface, forming a fine and uniform microcrack network. The connectivity of individual cracks is weakened, and the ductility of the specimen is significantly improved.

These results are fully consistent with the findings of “Aggregate edge number ranges: 8–15, 8–20, 8–25” section of this study regarding the effect of edge number and shape complexity. As the edge number range increases from 8–15 to 8–25, the aggregates transition from sharp, multi-angular morphologies to rounded, smoother profiles, and the failure mode shifts from sharp-corner-dominated concentrated shear to interface-dominated fine diffuse failure. Notably, although the volume fractions of the different edge number cases are similar and the differences in peak stress are small, a larger number of edges (i.e., a smoother profile) results in a more gradual post-peak stress decline and improved material ductility. This is fully consistent with the underlying mechanism by which polygonal aggregate morphology affects crack development and the brittle-ductile transition, as reported in the literature.

### Benchmark validation using uniaxial compressive stress–strain curves

**Fig. 11:**
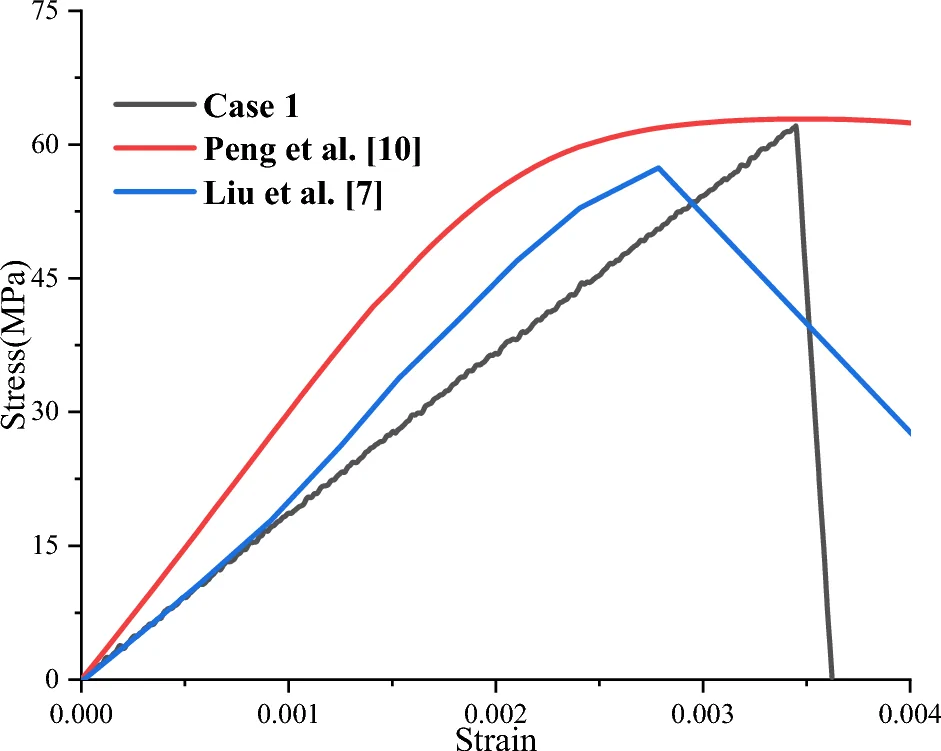
Comparison of uniaxial compressive stress–strain curves between the present simulation and existing studies.

To further verify the quantitative reliability of the proposed SPH model, the uniaxial compressive stress–strain curves from two representative studies are selected for comparison with the results of Case 1 of the present study, as shown in Fig. 11. Peng et al. (2019) considered dynamic loading conditions, while Liu et al. (2025) presented quasi-static experimental results for basic magnesium sulfate cement concrete (BMSCC). Both studies investigated the compressive mechanical behavior of concrete based on aggregate mesoscale models and can serve as effective validation benchmarks.

Comparison with Peng et al. (2019): That study investigated ordinary concrete and established a two-dimensional three-phase mesoscale model using the base force element method (BFEM). The specimen was represented as a two-dimensional cross-section of a 150 mm × 150 mm standard cube, with random spherical aggregates ranging from 5 to 40 mm generated via the Monte Carlo method and graded according to the Fuller distribution. The interfacial transition zone (ITZ) was explicitly modeled with independent mechanical parameters. The material parameters used in the present study are identical to those in that reference. In terms of loading, that study adopted displacement-controlled dynamic uniaxial compression and employed a multilinear two-dimensional damage constitutive model to describe the progressive damage evolution of each phase, resulting in a stress–strain curve characterized by a smooth pre-peak hardening segment and a gentle post-peak softening branch. In contrast, the present study adopts quasi-static loading without explicitly modeling the ITZ, yielding a curve with a steeper linear elastic rise and a sharp post-peak drop. The peak stresses and peak strains of the two models are generally close: the peak stress in Case 1 of the present study is 62.18 MPa, with a deviation of only 2.8% from the quasi-static results of Peng et al. (2019); the peak strain is 0.00345, with a relative deviation of less than 1.5%. The differences in curve shape mainly stem from the loading mode (dynamic vs. quasi-static), aggregate morphology (spherical vs. polygonal), interface treatment (explicit ITZ vs. equivalent interface), and damage description approach. Specifically, that study adopted a multilinear progressive damage constitutive model, where damage accumulates gradually, resulting in a smooth transition of the curve. In contrast, the present study employs a binary fracture model, in which the material loses its stiffness instantaneously once the strength threshold is reached, leading to a steep, nearly vertical curve shape. These differences represent reasonable methodological deviations and do not affect the validity of the mechanical trends.

Comparison with Liu et al. (2025): That study focused on basic magnesium sulfate cement concrete (BMSCC) and conducted quasi-static experiments and three-dimensional mesoscale simulations on 100 mm × 100 mm × 300 mm prismatic specimens. The aggregates were natural crushed stone with a continuous gradation of 5–16 mm. The material properties were as follows: elastic modulus of 23.62 GPa, Poisson’s ratio of 0.23, and average compressive strength of 57.58 MPa. The loading condition was quasi-static monotonic compression. The overall shape of the stress–strain curve is highly consistent with that of Case 1 in the present study. Owing to the high strength and high toughness characteristics of BMSCC, the curve of that study exhibits a longer linear elastic ascending segment and a relatively steeper initial post-peak descent. The present study adopts an aggregate compressive strength of 70 MPa and a cement matrix compressive strength of 32 MPa, also possessing relatively high strength characteristics, thus ensuring good comparability between the two studies. The minor differences between the two curves mainly arise from the model dimension (three-dimensional vs. two-dimensional) and aggregate morphology (natural crushed stone vs. artificial polygonal aggregates), which indirectly demonstrate that the proposed SPH model can effectively characterize the complete mechanical behavior of concrete under uniaxial compression.

In summary, through quantitative comparisons with the two aforementioned studies, the proposed SPH model exhibits good agreement with existing results in terms of stress–strain curve shape, peak stress, and peak strain, validating the reliability and applicability of the model.

## Discussion

### Verification of randomness

Since the aggregates in this study are generated using a stochastic algorithm, different random seeds for the same parameter set may produce different aggregate spatial configurations. To verify whether the observed trends are general rather than dependent on a specific aggregate arrangement, three representative cases (Case 1, Case 4, and Case 7) are selected. For each case, an additional independent random configuration (denoted with the suffix "-2") is generated and compared with the original configuration (denoted with the suffix "-1").

**Fig. 12:**
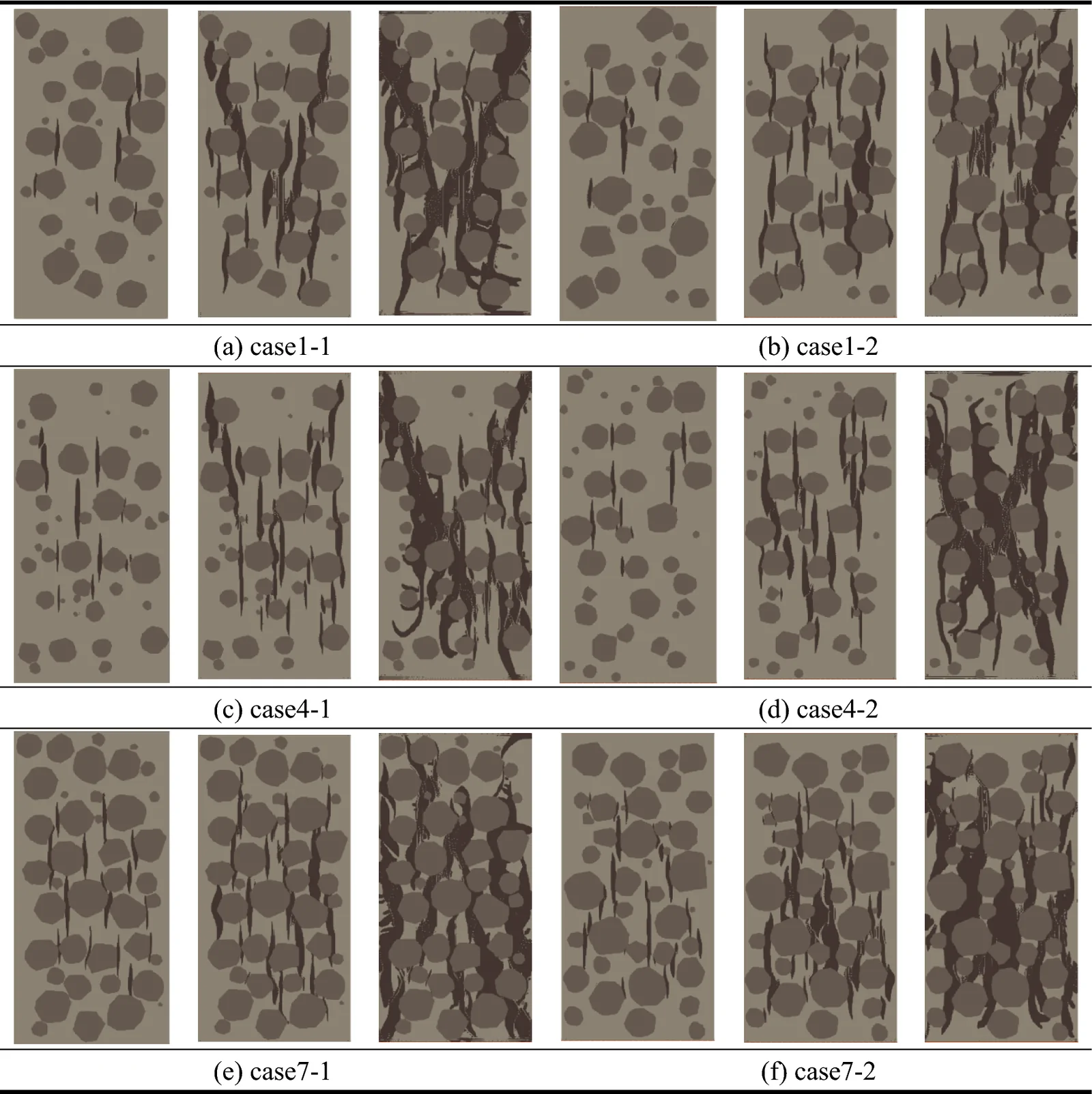
Comparison of crack evolution morphologies between two random realizations for representative cases: (**a**,**b**) Case 1; (**c**,**d**) Case 4; (**e**,**f**) Case 7.

Figure 12 compares the crack morphologies between the two random realizations. The results show that the crack initiation locations, propagation paths, damage distribution at the peak stage, and overall failure modes of Case 1–1 and Case 1–2, Case 4–1 and Case 4–2, and Case 7–1 and Case 7–2 are generally consistent, with no significant differences caused by the randomness of aggregate spatial distribution. This indicates that the observed regulatory effects of aggregate geometric characteristics on concrete failure modes exhibit good statistical stability and are not coincidental results arising from a specific random sample.

**Fig. 13:**
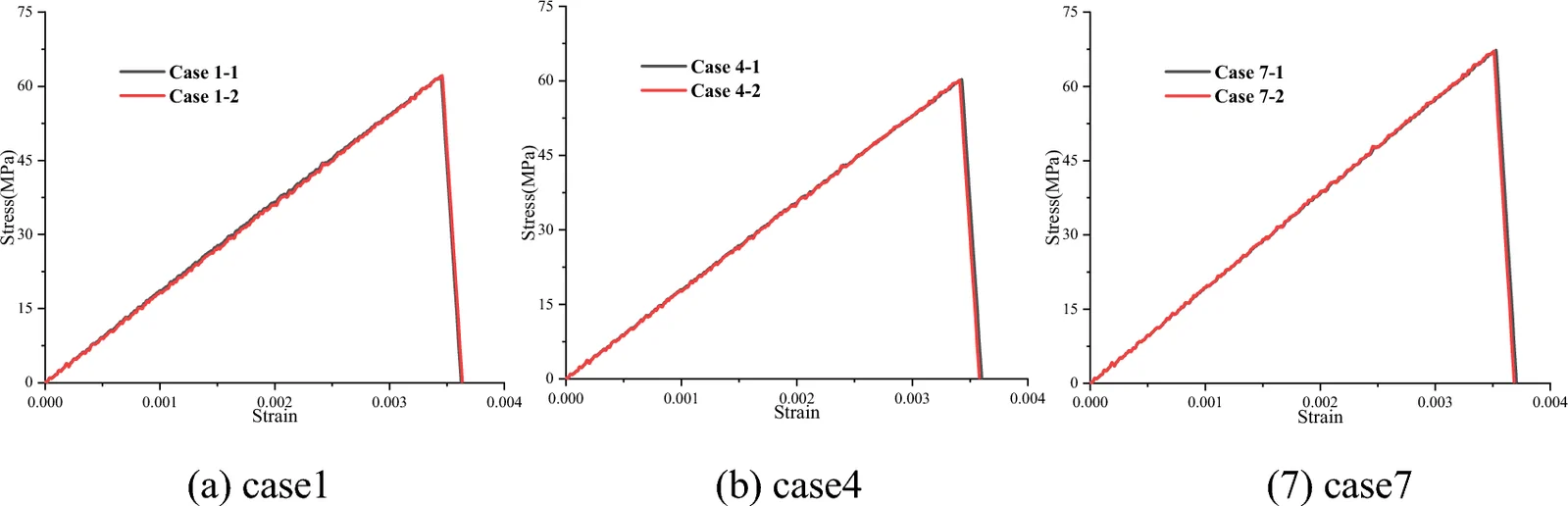
Comparison of uniaxial compressive stress–strain curves between two random realizations for representative cases: (**a**) Case 1; (**b**) Case 4; (**c**) Case 7.

Figure 13 presents a comparison of the corresponding stress–strain curves. The mechanical parameters for the two random realizations of each case are as follows: for Case 1, peak stress = 62.18 MPa (-1) and 62.31 MPa (-2), peak strain = 0.00345 and 0.00346; for Case 4, peak stress = 60.35 MPa (-1) and 60.28 MPa (-2), peak strain = 0.00343 and 0.00341; for Case 7, peak stress = 67.43 MPa (-1) and 67.31 MPa (-2), peak strain = 0.00353 and 0.00351. For each case, the two curves are essentially identical in shape, with peak differences of less than 0.3%, indicating good reproducibility of the computational results.

It is worth noting that in 2D mesoscale models, random aggregate arrangement is generally considered to have a significant influence on the mechanical response. However, in the three cases presented here, the deviations in mechanical parameters are all below 0.3%, with the stress–strain curves almost perfectly coincident. This apparent insensitivity can be attributed to the following factors.

First, the two random realizations under the same case have identical core parameters (aggregate number, size range, volume fraction), with only the aggregate positions differing. Consequently, the overall loading conditions are essentially consistent (the two models differ only in aggregate positions, while all other macroscopic geometric parameters remain identical), which fundamentally minimizes the differences in macroscopic mechanical response. Second, the research objective of this study is to investigate the influence of aggregate geometric characteristics (e.g., size, shape complexity) on concrete failure, rather than to analyze fluctuations caused by random positioning. The results show that differences between groups with different aggregate parameters are much larger than fluctuations within the same group, demonstrating that the observed trends possess good statistical stability. Third, different response quantities exhibit different sensitivity to aggregate positioning. Macroscopic indicators (peak stress, peak strain) are primarily governed by global conditions such as aggregate volume fraction and total interface area, where the influence of local position variations tends to be averaged out. Furthermore, the binary fracture model adopted in this study determines peak stress by the strength parameters of the matrix and aggregates rather than by crack propagation paths, further limiting the influence of local aggregate arrangement details on macroscopic load-bearing capacity. In contrast, local crack characteristics (initiation location, propagation path) are more sensitive to aggregate details—as shown in Fig. 12, while the two random realizations exhibit some differences in local crack details, their overall failure modes remain consistent.

In summary, the apparent insensitivity of the proposed model to random aggregate arrangement precisely demonstrates the good statistical stability of the revealed trends and clarifies the applicability scope of the model: it is suitable for investigating the effects of global aggregate statistical characteristics (e.g., size distribution, morphological parameters, gradation) on the macroscopic mechanical behavior of concrete.

### Advantages and limitations of the method

The advantages of this method are as follows: it achieves integrated construction of parametric automatic modeling of random polygonal aggregates and SPH failure simulation; it achieves accurate collision detection of polygons based on the separating axis theorem; and it achieves precise classification of particle attributes based on the ray casting method, avoiding the mesh dependence problem of the traditional finite element method.

The limitations of this study include the following: (1) Currently, only a two-dimensional polygonal aggregate model is considered, whereas aggregates in actual concrete exhibit three-dimensional irregular morphologies. Two-dimensional simulations are adopted in this study for parametric comparative analysis, as they allow efficient generation of a large number of random configurations, which is a common practice in mesoscale concrete modeling^10,13^. Furthermore, two-dimensional simulations eliminate the interference of out-of-plane geometric uncertainties, allowing a clearer identification of the pure mechanistic influence of aggregate morphology on crack propagation. Although absolute stress values may differ under three-dimensional conditions, the key qualitative trends revealed in this paper—such as the transition from localized shear to diffuse damage with increasing aggregate density, or the improvement in ductility with increasing shape complexity—are governed by fundamental mechanisms (stress concentration at aggregate corners and aggregate-matrix interface interaction) that are dimension-insensitive and are expected to hold under three-dimensional conditions. Future work will extend the method to three dimensions to quantify the effects of out-of-plane aggregate geometry. (2) A homogenized material assumption is adopted, and the aggregate-matrix interfacial transition zone (ITZ) is not explicitly modeled. The ITZ is a weak region around aggregates and is a preferential location for crack initiation. The omission of explicit ITZ modeling may lead to underestimation of early-stage crack initiation density. However, this simplification does not affect the reliability of the comparative conclusions of this study, because the focus is on the qualitative trends of failure modes governed by aggregate geometric characteristics rather than on absolute cracking loads. Moreover, the Mohr–Coulomb criterion adopted for the matrix phase implicitly captures the weak interface behavior to some extent (cracks preferentially initiate and propagate at aggregate corners and boundaries). Furthermore, as a parametric comparative study, the omission of the ITZ affects all cases equally, and thus the relative trends remain reliable. Nevertheless, we acknowledge that explicit ITZ modeling would contribute to more precise predictions of crack initiation locations and load levels. Future work will introduce an independent ITZ phase to achieve more quantitative predictions. (3) Only the uniaxial compression condition is considered, and multiaxial stress states are not addressed. In practical engineering, concrete is often subjected to complex stress conditions, under which the failure mechanism may differ from that under uniaxial compression. Therefore, extending the proposed method to multiaxial stress conditions is an important direction for future work. (4) Additionally, this study does not provide a systematic sensitivity analysis of the constitutive model parameters. All material parameters are taken from Ref.^10^, and the same parameters are used for all cases. Although this setup ensures the comparability of the relative effects of aggregate geometric parameters, the potential influence of parameter selection on absolute strength predictions has not been quantified. Parameter sensitivity remains an open question for future research. (5) The binary fracture formulation used in this work cannot describe the progressive damage evolution of concrete during compression. Accordingly, the simulated stress–strain curves present nearly linear pre-peak responses and abrupt post-peak drops, which deviate from the nonlinear hardening and gradual softening behaviours observed in real concrete. For further improvement, continuous damage models incorporating stiffness degradation or evolving damage factors can be implemented. Such modifications are expected to better reproduce the pre-peak nonlinearity and post-peak softening characteristics without compromising the identification of crack propagation paths, thus producing numerical results closer to experimental measurements.

## Conclusions

1. This study successfully implements an SPH-based simulation framework for uniaxial compression failure of concrete with random polygonal aggregates. The framework integrates the Mohr–Coulomb criterion with tensile cutoff and an improved smoothing kernel function to capture spontaneous crack initiation and propagation. It also incorporates a self-developed particle generation program, based on the separating axis theorem and ray casting method, to automatically construct aggregate-matrix separated SPH models, providing an efficient platform for mesomechanical studies of concrete.
2. Aggregate density and size distribution are key factors controlling the failure mode and brittleness of concrete. As the number of aggregates increases from 30 to 50, the crack area ratio at peak stress increases from 7.2 to 14.8% (more than doubling), and the failure mode transitions from localized shear to diffuse damage patterns. Similarly, expanding the size range from 1–8 mm to 1–12 mm increases the crack area ratio from 4.8 to 13.2%, accompanied by a transition toward more distributed failure. These quantitative indicators provide evidence for enhanced material brittleness with increasing aggregate density or size range.
3. In contrast, aggregate shape complexity improves material ductility. When the side number range increases from 8–15 to 8–25, the failure mode shifts from sharp corner-dominated concentrated shear to interface-dominated fine diffuse failure. Although the peak stress varies only slightly (about ± 1.6%), the post-peak curve morphology differs markedly, and the crack pattern becomes more uniformly distributed, indicating improved ductility.
4. Quantitative comparisons with existing experimental and numerical studies show good agreement in terms of stress–strain curve shape, peak stress, and peak strain, validating the reliability of the proposed SPH model for simulating the mesomechanical behavior of concrete with irregular aggregates.

## Data Availability

The datasets generated and/or analysed during the current study are available from the corresponding author upon reasonable request.
